# Supplementary material for: Accurate and scalable variant calling from single cell DNA sequencing data with ProSolo
Source: Nat Commun. 2021 Nov 18;12:6744. doi: 10.1038/s41467-021-26938-w (PMC8602313; doi:10.1038/s41467-021-26938-w)
Supplement: Supplementary file 1 — Supplementary Information [file 41467_2021_26938_MOESM1_ESM.pdf]

Accurate Variant Calling from Single Cell DNA Sequencing Data with ProSolo

Supplementary Information

David Lähnemann, Johannes Köster, Ute Fischer,
Arndt Borkhardt, Alice C. McHardy, Alexander Schönhuth

November 10, 2021

Contents

1 Supplementary Note 1: Probabilistic model of ProSolo 2
1.1 Underlying True Alternative Nucleotide Frequencies: θ 2
1.2 Non-reference Nucleotide Frequencies After Biased Whole Genome Amplification: ρ 3
1.2.1 The Pólya urn model: motivating the use of beta-binomial distributions 6
1.2.2 Outlook: more realistic beta-binomial distributions 9
1.3 Reads: Sampling Alternative Nucleotide Frequencies in the Sequencing Process 10
1.4 Single cell latent variable model 13
1.5 Bulk background: Accounting for Amplification Errors and Allele Dropout . 17
1.5.1 Defining the Event Space 17
1.5.2 Posterior Probabilities of Events 21

1.6 Implementation 24
2 Supplementary Note 2: Benchmarking 26
2.1 Datasets and Ground Truths 26
2.2 Software and Parameters 35
2.3 Alternative Allele Calling 39
2.4 Estimating the bulk contribution to ProSolo results via downsampling . . . 48
2.5 Recall of validated somatic variants from Wang et al. [48] 48
2.6 False Discovery Rate Control of Alternative Allele Calling 53
2.7 Allele dropout rate calculations 56
2.8 Genotype calling 59
2.9 Software and Resources used 63

1 Supplementary Note 1: Probabilistic model of ProSolo

1.1 Underlying True Alternative Nucleotide Frequencies: θ

The eventual goal in variant calling is to infer the true frequency of a possible alternative, and thus variant, nucleotide in the sample you sequenced. For such an alternative nucleotide $x \in \{A, C, G, T\}$ at a particular site in a genome, we therefor define[a]:

$$\theta_b(x), \theta_s(x) \tag{S 1}$$

as the true (but usually unknown) frequency of alternative nucleotide x in the bulk and single cell sample, respectively. These frequencies translate into probabilities of sampling a DNA fragment containing the respective nucleotide at a site: either directly, as with the sequencing of DNA from a bulk of cells b; or indirectly, as with the sequencing of whole genome amplified DNA of a single cell s. It is our eventual goal to work back from bulk and single cell data to derive likelihoods for all possible values of $\theta_b(x)$ (denoted

[a]We omit an explicit notation for a site, but every θ is always specific to one genome position.

as $\tilde{\theta}_b(x)$)[b] and $\theta_s(x)$ (denoted as $\tilde{\theta}_s(x)$), respectively, allowing us to place likelihoods on possible underlying genotypes and events like allele dropouts in single cells.

For a single diploid cell s, the true alternative nucleotide frequency can be

$$\theta_s(x) = \begin{cases} 0 \\ \frac{1}{2} \\ 1 \end{cases} \tag{S 2}$$

reflecting the cases that: (i) x is not present at the position—if no other allele is present, this constitutes a homozygous reference site; (ii) x reflects an alternative nucleotide that is present in only one of the two genome copies of the single cell—this constitutes a heterozgyous site; (iii) x is an alternative nucleotide present in both genome copies of the single cell—this constitutes a homozygous alternative site.

Bulk experiments contain subpopulations that can differ in their genotype regarding a particular site, through a somatic mutation that one of the subpopulations has acquired in one allele. They can thus exhibit any alternative nucleotide frequency ($\theta_b(x) \in [0, 1]$), depending on the genotypes present and their relative abundance (Supplementary Figure 2).

1.2 Non-reference Nucleotide Frequencies After Biased Whole Genome Amplification: ρ

Let further $\rho_s(x), \rho_b(x)$ be the abundance of genome copies showing alternative nucleotide x at a particular site in the single or bulk DNA sample, respectively, right before the sequencing—for the single cell, this means after the whole genome ampli-

[b] We use θ notation for the true underlying alternate allele frequency at a site, and $\tilde{\theta}$ notation when referring to the likelihood density estimates derived for all possible underlying alternate allele frequencies. The latter notation is thus mostly used for the definition of single cell events in section 1.5.1.

fication that is necessary to obtain enough DNA for sequencing with technologies like Illumina. These abundances translate into probabilities to draw a read containing x from the respective sample (in the sequencing experiment), given that such a read covers the genome position.

Under the assumption of uniform abundance of all parts of the genome and uniform abundance of each haplotype, which is reasonable in bulk sequencing (i.e. without amplification), it holds that $\rho = \theta$, so we henceforth assume that

$$\rho_b(x) = \theta_b(x). \tag{S 3}$$

In contrast, in single cell sequencing—due to the necessary whole genome amplification process—the assumption of uniform abundance of all parts of the genome and of each haplotype does not hold. Instead, each allele at a particular site can be amplified to a different degree, ranging from no amplification at all (i.e. a complete allele dropout) to several hundred or thousand copies. Thus

$$\rho_s(x) \neq \theta_s(x) \quad \text{but instead} \quad \rho_s(x) \sim P(\rho_s \mid \theta_s(x)) \tag{S 4}$$

that is, ρ_s is a random variable governed by a probability distribution $P(\rho_s \mid \theta_s(x))$ that depends on the original alternative nucleotide frequency θ_s. $P(\rho_s \mid \theta_s(x))$ reflects the differential amplification of the two alleles, which is only detectable for heterozygous sites ($\theta_s = \frac{1}{2}$), thus

$$P(\rho_s \mid \theta_s(x)) = \begin{cases} P(\rho_s \mid \theta_s(x) = 0) \\ P(\rho_s \mid \theta_s(x) = \frac{1}{2}) \\ P(\rho_s \mid \theta_s(x) = 1) \end{cases} \tag{S 5}$$

Understanding these probability densities is key to understanding the statistical issues

single cell whole genome amplification methods come along with[c]. From the Supplementary Material of Lodato et al. [28] (see Figure S5 and the section "Modeling MDA-derived alternative read counts" of the respective supplement for the details), we get an empirical model that characterizes them for the whole genome amplification method multiple displacement amplification (MDA)[d]:

$$\begin{aligned} P(\rho_s \mid \theta_s = 0) &= \mathfrak{B}\mathfrak{B}_{l,\alpha,\beta}(\rho_s) \\ P(\rho_s \mid \theta_s = \frac{1}{2}) &= w(l) \times \mathfrak{B}\mathfrak{B}_{l,\alpha_1,\alpha_1}(\rho_s) + (1 - w(l)) \times \mathfrak{B}\mathfrak{B}_{l,\alpha_2,\alpha_2}(\rho_s) \\ P(\rho_s \mid \theta_s = 1) &= \mathfrak{B}\mathfrak{B}_{l,\beta,\alpha}(\rho_s) \end{aligned} \tag{S 6}$$

where $\mathfrak{B}\mathfrak{B}$ represents beta-binomial functions with the parameters linearly scaled by l, the total observations at a site (i.e. the total read coverage). For homozygous reference sites ($\theta_s = 0$), the parameters of the beta-binomial function are $\alpha(l)$ and $\beta(l)$. For the heterozygous sites ($\theta_s = \frac{1}{2}$), both beta-binomial functions are symmetrical with $\alpha_1(l), \alpha_2(l)$ dependent on the site-specific coverage l and combined by factor $w(l)$. $\rho_s \in \frac{k}{l}, k \in 0, 1, \ldots, l$ are the possible cases of alternative nucleotide frequencies. In their Supplementary Figure S5 in panels C and D, Lodato et al. [28] give estimates of the parameters $\alpha(l), \beta(l), w(l), \alpha_1(l), \alpha_2(l)$ obtained by fitting to empirical data. We list the values we extracted in Table 1. For the third case (homozygous alternative nucleotide, i.e. $P(\rho_s \mid \theta_s = 1)$) we mirror the homozygous reference function[e].

Using one of the possible expressions of the respective probabilities (Equation 6.28

[c] Note, in addition to the whole genome amplification bias, these distributions can implicitly also model the amplification errors that the polymerases used for whole genome amplification introduce in the copy process.

[d] For other whole genome amplification methods with different amplification bias properties, we can simply implement a different instance of the general model for $P(\rho_s \mid \theta_s(x))$ accordingly. And for MDA, different empirical models learned from other or more published datasets could be implemented as well as strategies of inferring parameters from the very dataset analysed in a certain run of the software.

[e] Note that the amount of sites that are homozygous for the reference allele is much larger, which also means that statistics for $\theta_s = 1.0$ are more robust when being derived from the case $\theta_s = 0.0$.

in Section 6.2.4 of Johnson et al. [18])[f], with regards to the beta function $\mathfrak{B}$, we can compute the probability of observing k reads with the alternative nucleotide at a site of coverage l for the heterozygous case as follows:

$$P(K = k \mid \theta_s = \frac{1}{2}) = w(l) \times \binom{l}{k} \frac{\mathfrak{B}_{k+\alpha_1, l-k+\alpha_1}}{\mathfrak{B}_{\alpha_1,\alpha_1}} + (1 - w(l)) \times \binom{l}{k} \frac{\mathfrak{B}_{k+\alpha_2, l-k+\alpha_2}}{\mathfrak{B}_{\alpha_2,\alpha_2}} \tag{S 8}$$

In the future, these distributions could be learned from the dataset at hand, whenever large enough sets of sites that have a high probability of being homozygous reference or heterozygous are available. In addition, the exact distribution used could be further refined with the below considerations regarding the suitability of beta-binomial distributions in this case.

1.2.1 The Pólya urn model: motivating the use of beta-binomial distributions

For the heterozygous case, the applicability of the beta-binomial distribution [17] should be immediately evident from the formulation of the Pólya urn model [12] that generates beta-binomial distributions [17, 18]. It works directly analogous to the amplification

[f] Here, the parameters b, w, c from the formulation in Equation 6.28 in Section 6.2.4 of Johnson et al. [18] are summarised into α_1, α_2 from Lodato et al. [28] with:

$$\alpha_1 = \frac{w_1}{c} = w_1 = b_1 \qquad \alpha_2 = \frac{w_2}{c} = w_2 = b_2 \tag{S 7}$$

I.e., the number of starting reference and alternative alleles is identical within each beta-binomial, yielding symmetric distributions. In terms of the Pólya urn model used as motivation in Johnson et al. [18], this equates to the number of w white and b black balls being identical. c, the number of balls of identical color that are added whenever a ball of particular color is added, is set to 1. Further considerations for obtaining the values of b, w are discussed below in relation to the Pólya urn model.

process[g]:

At the start, the urn contains w $w$hite and b $b$lack balls, analogous to the two alleles present at a heterozygous site—let us say white balls represent the reference allele and black balls the alternative allele. Whenever a ball is drawn, it is replaced to the urn and another c balls of the same $c$olor are added, analogous to the polymerase copying an allele.

As we have two strands each for both alleles, a natural choice of starting alleles would be $w_t = b_t = 2$. We here subscript the values with $_t$ to denote the theoretical nature of the parameter choice, as opposed to practical values of w_p, b_p introduced below. And as one strand is added with every copy operation, we set $c = 1$ (Supplementary Figure 1A). This gives us a slight peak around the alternative allele frequency of 0.5 (Supplementary Figure 1A), which we can also make out in the real data (Figure 3B of the main manuscript).

However, it does not capture the allele dropout peaks at the allele frequencies of 0 and 1 seen there. A likely reason for these dropout peaks is, that not every position on every allele is equally accessible to amplification initiation [39]. I.e. mechanistically, some close-by site to the one under consideration must be single-stranded at some point during the continuous amplification reaction (at a constant temperature), to be accessible for an initial priming that will start the amplification of the respective allele for that region. To account for this lower initiation accessibility, we can introduce a scaling factor f for the starting frequencies of the two alleles, with $0 <= f <= 1$, to scale down the initially available allele count: if strands are already separated for this allele and amplification initiation works perfectly for both strands ($f = 1$), then we would get $w_p = w_t * f = 2 * 1 = 2$; whereas at the other extreme of $f = 0$, amplification initialisation of the allele would be impossible. Then, for $f = \frac{1}{2}$, we get the uniform distribution ($w_p = w_t * f = \frac{1}{2} * 2 = 1$, Supplementary Figure 1B), and for all $f < \frac{1}{2}$, we

[g]The notation used here is analogous to Equation 6.28 in Section 6.2.4 of Johnson et al. [18]. Also see Equation S 7 in the above footnote for its relation to α_1, α_2 in Lodato et al. [28].

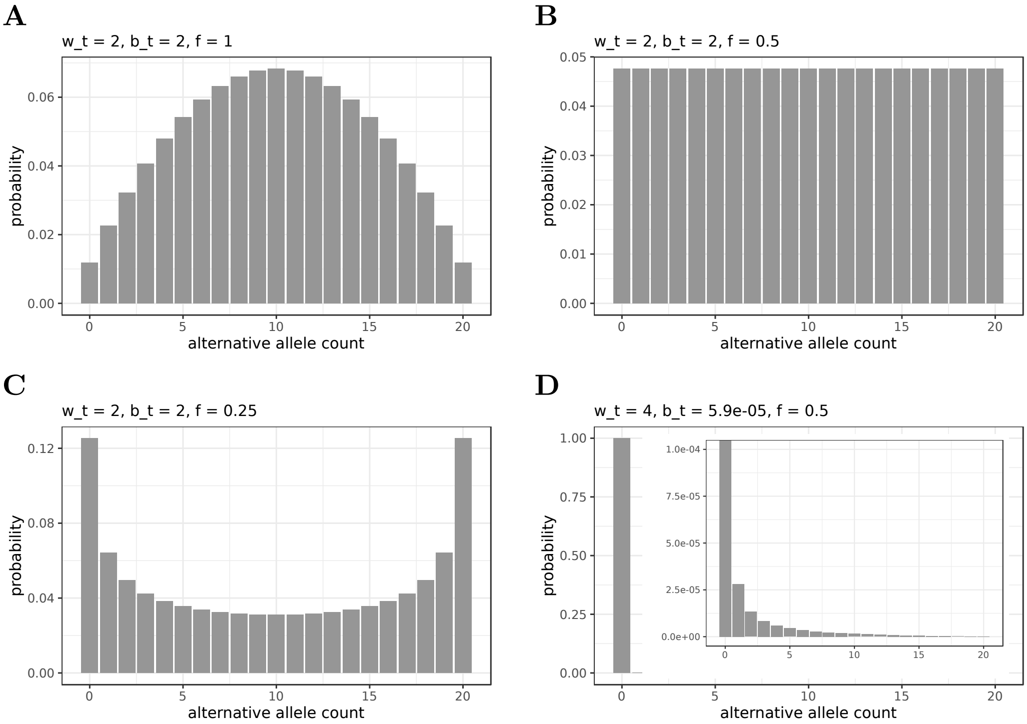

Supplementary Figure 1: Beta-binomial distributions with different shape parameters, that correspond to our theoretical numbers of starting allele strands ($w_t = b_t = 2$) scaled to different amplification accessibility by f. **A** Perfect accessibility of both strands of both alleles for amplification ($f = 1$) and the resulting alternative allele frequencies. **B** Half that accessibility ($f = 0.5$), resulting in the uniform distribution. **C** For any accessibility scaling value below $\frac{1}{2}$ (e.g. $f = 0.25$), the beta-binomial distribution will give maxima at alternative allele frequencies 0 and 1. **D** The heavily non-symmetrical case ($w_t \gg b_t$), analogous to homozgyous reference genotypes. Instead of the distribution currently used in ProSolo, which was fit to data and compounds sequencing errors with amplification errors (Figure 3A in the main manuscript), this distribution makes the following theoretical assumptions: It sets $w_t = 4$ for the 4 reference strands available for amplification and b_t to four times the known $\Phi 29$ error rate for drawing / introducing an error alternative allele instead of the reference allele. The inset zooms into the y-axis to show details of the tail of the distribution.

will get distributions with peaks at 0 and 1 (e.g. Supplementary Figure 1C).

If we then consider a mixture of sites with different f, we would expect distributions as observed e.g. in Figure 3B of the main manuscript; and a reasonable approximation of this is the mixture of the two symmetrical beta-binomials as fitted by Lodato et al. [28], one with $f \approx 0.40 < \frac{1}{2}$ and one with $f \approx 0.67 > \frac{1}{2}$ (i.e. the intersects of α_2 and α_1 plus their slight slope-adjustments, as given in Table 1).

1.2.2 Outlook: more realistic beta-binomial distributions

However, something that this does not capture is possible asymmetry of the distributions, i.e. the case of $w \neq b$. While the theoretical starting amounts of strands of both alleles in the heterozygous case should remain the same ($w_t = b_t = 2$), we would have to introduce allele-specific scaling factors $f_w \neq f_b$ that capture the different availability of the two alleles for amplification (e.g. because one of the alleles is more densely wrapped around Histones than the other). As a result we would get $w_p = w_t * f_w \neq f_b * b_t = b_p$, and instead of the one shape parameter in the symmetrical case, we would then have to fit the distributions with a second shape parameter. I.e. instead of Equation S 7, we would get:

$$\begin{aligned} \alpha_1 &= w_p = w_t * f_w = 2f_w \\ \beta_1 &= b_p = b_t * f_b = 2f_b \end{aligned} \tag{S 9}$$

However, assuming that the dropout of both alleles—reference and alternative—is equally likely, this asymmetry will average out over all heterozygous sites, and the symmetry assumption remains a justified reduction in parameters to fit when fitting a mixture of two beta-binomials.

This is somewhat different for the homozygous case. Here, Lodato et al. [28] already

modelled the allele frequency deviations (> 0 for homozygous reference) as an asymmetric beta-binomial distribution. This gives a reasonable fit to the data and makes sense to use, as we would expect these deviations to be the result of amplified polymerase errors from the MDA. While we just use the distributions fit by Lodato et al. [28] in our initial implementation we present here, this could in the future be replaced by a theoretically derived beta-binomial distribution. For example, for the homozygous reference case, a very low b_t (the probability of “choosing” a previously non-existing error allele) could be determined by (four times) the known $\Phi 29$ polymerase error rate, whereas w_t would be set to 4 to account for four reference strands available for copying (Supplementary Figure 1D). As f would in this case affect both the reference (w) and the error allele (b) alike, it could be set to some identical value for both, e.g. to an average accessibility of 0.5 (Supplementary Figure 1D). This would avoid compounding the uncertainty due to MDA with sequencing errors, that we already account for separately in our latent variable model through base calling quality scores and is one very likely source of the current mis-classifications of ground truth heterozygous sites as homozygous (Supplementary Figure 16).

1.3 Reads: Sampling Alternative Nucleotide Frequencies in the Sequencing Process

Let $\mathfrak{R}$ ($=\mathfrak{R}(g)$) denote all reads covering a site g, with $R_i \in \mathfrak{R}$ denoting an individual read out of the l reads aligning to that site ($l \in \mathbb{N}, i \in \{1, 2, \ldots, l\}$). Formally, we index positions in aligned reads by the respective genome indices g, but we omit the index g for readability. Working with a given ρ, one can assume independence among the reads in the respective sample (while, of course, given θ, one can assume independence only in bulk, due to the necessary amplification and the resulting coverage nonuniformity in single cell samples). That is

$$L(\rho \mid \mathfrak{R}) \propto P(\mathfrak{R} \mid \rho) = \prod_{R \in \mathfrak{R}} P(R \mid \rho) \propto \prod_{R \in \mathfrak{R}} L(\rho \mid R) \tag{S 10}$$

both for bulk and single cell sequencing. Let $S_{R_i}[g]$ be the nucleotide determined by the sequencer for position g of read R_i and $Q_{R_i}[g]$ be the Phred quality score for the nucleotide at that position of read R_i[h]. Let further $M_{R_i}[g]$ be the Phred alignment quality score[i] provided by the aligner. We now define $Z_i[g]$ ($i \in \{1, \ldots, l\}$) to be the collection of the observable variables for the alignment of one of l reads $R_i \in \mathfrak{R}$ to a genome position g. Omitting index g, this is the vector $Z_i = \langle S_{R_i}, Q_{R_i}, M_{R_i} \rangle$ and drawing on this information, we now introduce hyperparameters analogously to the model for tumor-normal variant calling as outlined by Köster et al. [23] for Varlociraptor. These will be the latent variables $\omega_i \in \{0, 1\}$ to model correct placement of an alignment ($\omega_i = 1$ if correct and $\omega_i = 0$ otherwise) and $\xi_i \in \{0, 1\}$ to specify whether an alignment Z_i is associated with the alternative nucleotide ($\xi_i = 1$) or not ($\xi_i = 0$). Taken together, they give three cases

$$(\omega_i, \xi_i) = \begin{cases} (1,1) : R_i \text{ mapped correctly, alternative nucleotide} \\ (1,0) : R_i \text{ mapped correctly, reference nucleotide} \\ (0,\{0,1\}) : R_i \text{ not mapped correctly, nucleotide irrelevant} \end{cases} \tag{S 11}$$

We thus model the two major sources of uncertainty on the level of the individual alignment Z_i, 1) *alignment uncertainty* and 2) *typing uncertainty*, by associating every

[h] As above, we now omit g for S_{R_i} and Q_{R_i} for ease of notation and reading, as we will for M_{R_i}. Also note that the value of $Q_{R_i}[g]$ can be adjusted by a base quality score recalibration that accounts for systematic base calling errors, before using it as input to ProSolo.

[i] Also called mapping quality; represented by the MAPQ tag in the sequence alignment (SAM) format specifications v1 (2a802cd) and by the MQ tag in the variant call format (VCF) specification v4.3 (2a802cd). As before, we will omit the position g from notation from this point on.

alignment Z_i with these two binary-valued, latent variables, reflecting uncertainty hyperparameters, ω_i and ξ_i:

First,

$$\omega_i \sim \text{Bernoulli}\left(\pi_i\right) \;\; \text{for } i = 1, \ldots, l \tag{S 12}$$

where π_i is the (Phred scaled) posterior probability proportional to M_{R_i} provided by the aligner. We can compute it with

$$\pi_i = 10^{-M_{R_i}/10} \tag{S 13}$$

Second,

$$\xi_i \sim \text{Bernoulli}\left(\rho\right) \;\; \text{for } i = 1, \ldots, l \tag{S 14}$$

which reflects that sampling a fragment from the locus that bears the alternative nucleotide agrees with the probability to sample a genome copy with the alternative nucleotide from the pool of amplified DNA.

Whether ξ_i is 1 or 0 is generally not evident from the observed Z_i, due to typing uncertainty. We formally define[j]

$$Z_i \mid \omega_i = 1, \xi_i = 0 \sim a(Z_i) \quad \text{and} \quad Z_i \mid \omega_i = 1, \xi_i = 1 \sim p(Z_i) \tag{S 15}$$

where a, p are probability distributions over correct S_{R_i} when the alternative nucleotide is either *a*bsent or *p*resent in read R_i and $a(\cdot)$ and $p(\cdot)$ are the respective functions to sample from this distribution for a particular read. With these probability distributions, we can account for any number of factors for which we know how they

[j] If $\omega_i = 0$, that is the alignment is incorrect, $Z_i \mid \omega_i = 0 \sim 1$ reflects that Z_i has no influence on the posterior probability distribution of ρ.

affect these probability distributions—but if we lack any further information for an alternative nucleotide, we can assume Q_{R_i} to be a specification of these distributions for R_i, accounting for the uncertainties of the sequencing process. With $x \in \{A, C, G, T\}$ the alternative nucleotide under inspection, we can thus compute:

$$a(Z_i) = \begin{cases} 10^{-Q_{R_i}/10} & \text{if } S_{R_i} = x \\ 1 - 10^{-Q_{R_i}/10} & \text{if } S_{R_i} \neq x \end{cases} \tag{S 16}$$

$$p(Z_i) = 1 - a(R_i) \tag{S 17}$$

1.4 Single cell latent variable model

By applying the Chapman-Kolmogorov equation with our binary hyperparameters to the single cell case[k], we get

$$L(\rho_s \mid Z_i^s) \propto \; P(Z_i^s \mid \rho_s) = \int_{\omega_i^s, \xi_i^s} P(Z_i^s \mid \omega_i^s, \xi_i^s) \times P(\omega_i^s, \xi_i^s \mid \rho_s)\, d(\omega_i^s, \xi_i^s) \tag{S 18}$$

which fans out to the following cases from equation S 11:

$$\begin{aligned} P(Z_i^s \mid \rho_s) = P(Z_i^s \mid \omega_i^s = 0) &\times P(\omega_i^s = 0 \mid \rho_s) \\ + P(Z_i^s \mid \omega_i^s = 1, \xi_i^s = 0) &\times P(\omega_i^s = 1, \xi_i^s = 0 \mid \rho_s) \\ + P(Z_i^s \mid \omega_i^s = 1, \xi_i^s = 1) &\times P(\omega_i^s = 1, \xi_i^s = 1 \mid \rho_s) \end{aligned} \tag{S 19}$$

Knowing that the mapping probability of reads is independent from the given (distorted) allele frequency ρ_s, and assuming a sampling probability of 1 for any mismapped

[k] We will only be looking at single cell samples in the following, as the case of $\rho_b = \theta_b$ is the simpler one and analogous without the whole genome amplification considerations. Please also refer to the tumor and normal sample derivations in Köster et al. [23], but note that we need no purity parameter, here.

read, this becomes:

$$
\begin{aligned}
P(Z_i^s \mid \rho_s) = P(\omega_i^s = 0) & \\
+ P(Z_i^s \mid \omega_i^s = 1, \xi_i^s = 0) \times P(\omega_i^s = 1) \times P(\xi_i^s = 0 \mid \rho_s) & \\
+ P(Z_i^s \mid \omega_i^s = 1, \xi_i^s = 1) \times P(\omega_i^s = 1) \times P(\xi_i^s = 1 \mid \rho_s) &
\end{aligned} \tag{S 20}
$$

With Equation S 12 for ω_i^s, Equation S 14 for ξ_i^s and Equations S 16 and S 17 for the conditional probabilities of the data given an absence or presence of the observed nucleotide, we get:

$$
\begin{aligned}
P(Z_i^s \mid \rho_s) = (1 - \pi_i^s) & \\
+ \; a^s(Z_i^s) \times \pi_i^s \times (1 - \rho_s) & \\
+ \; p^s(Z_i^s) \times \pi_i^s \times \rho_s &
\end{aligned} \tag{S 21}
$$

As the l reads are conditionally independent, given a particular (distorted) alternative nucleotide frequency ρ_s (also see Equation S 10), we can calculate the likelihood of such a particular ρ_s with:

$$
L(\rho_s \mid \boldsymbol{Z}^s) \propto P(\boldsymbol{Z}^s \mid \rho_s) = \prod_{i=1}^{l} P(Z_i^s \mid \rho_s) \tag{S 22}
$$

With the resulting likelihoods for $\rho_s \in [0, 1]$, we can calculate the likelihood of a particular θ_s:

$$
L(\theta_s \mid \boldsymbol{Z}^s) \propto P(\boldsymbol{Z}^s \mid \theta_s) = \sum_{k=0}^{l} \left\{ P(\boldsymbol{Z}^s \mid \rho_s = \frac{k}{l}) \times P(\rho_s = \frac{k}{l} \mid \theta_s) \right\} \tag{S 23}
$$

For $\theta_s \in \{0, \frac{1}{2}, 1\}$, with equation S 5 specifying the cases of θ_s and Equation 2 further specifying $P(\rho_s \mid \theta_s)$ for MDA, this gives us:

Supplementary Table 1: Parameters of the multiple displacement amplification (MDA) amplification bias model as presented in Supplementary Figure S5, panels C and D of Lodato et al. [28]. Each of these scales linearly up to a site coverage of 60, with the slopes and intercepts as given here.

parameter	slope	intersect
α	-0.000027183	0.068567471
β	0.007454388	2.367486659
w	0.000548761	0.540396786
α_1	0.057378844	0.669733191
α_2	0.003233912	0.399261625

$$
\begin{aligned}
P(\boldsymbol{Z}^s \mid \theta_s = 0) &= \sum_{k=0}^{l} \left\{ P(\boldsymbol{Z}^s \mid \rho_s = \frac{k}{l}) \times \mathfrak{BB}_{l,\alpha,\beta}(\rho_s = \frac{k}{l}) \right\} \\
P(\boldsymbol{Z}^s \mid \theta_s = \frac{1}{2}) &= \sum_{k=0}^{l} \Big\{ P(\boldsymbol{Z}^s \mid \rho_s = \frac{k}{l}) \times \Big[w(l) \times \mathfrak{BB}_{l,\alpha_1,\alpha_1}(\rho_s = \frac{k}{l}) + \\
&\qquad (1 - w(l)) \times \mathfrak{BB}_{l,\alpha_2,\alpha_2}(\rho_s = \frac{k}{l}) \Big] \Big\} \\
P(\boldsymbol{Z}^s \mid \theta_s = 1) &= \sum_{k=0}^{l} \left\{ P(\boldsymbol{Z}^s \mid \rho_s = \frac{k}{l}) \times \mathfrak{BB}_{l,\beta,\alpha}(\rho_s = \frac{k}{l}) \right\}
\end{aligned}
\tag{S 24}
$$

For the current implementation, we use the empirical values for $\alpha(l), \beta(l), w(l), \alpha_1(l), \alpha_2(l)$ as deduced by Lodato et al. [28] (Supplementary Table 1). But as mentioned above, these could be learned from a dataset at hand whenever high-confidence sets of heterozygous and homozygous sites are available (e.g. by calling variants on a background bulk and cross-referencing with dbSNP).

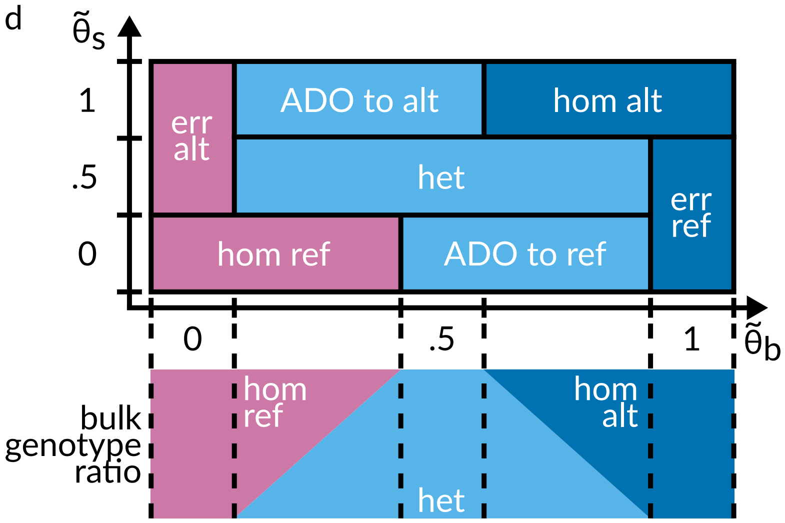

Supplementary Figure 2: It depicts single cell events at a particular genomic site, defined by combinations of ProSolo's maximum likelihood estimates for possible true alternative nucleotide frequencies in single cell sequencing ($\tilde{\theta}_s$) and bulk sequencing samples ($\tilde{\theta}_b$) in our probabilistic model. The bulk is always assumed to be a combination of a maximum of two genotypes at a particular site, generating all possible $\tilde{\theta}_b$ (bottom panel). A tabular representation of the same event definitions is given in Supplementary Table 2 below (this is a larger copy of Figure 3D in the main manuscript, reproduced here for easier cross-reference with that table).
ADO – allele dropout, alt – alternative, err – error, het – heterozygous, hom – homozygous, ref – reference

Supplementary Table 2: Single cell events at a particular genomic site are defined by combinations of ProSolo's likelihood density estimates of the possible true alternative nucleotide frequency ranges in single cell sequencing ($\tilde{\theta}_s$) and bulk sequencing samples ($\tilde{\theta}_b$) in our probabilistic model. A graphical representation of the same event definitions is given in Supplementary Figure 2.

single cell event	$\tilde{\theta}_s(x)$	$\tilde{\theta}_b(x)$	implicit single cell genotype
$\boldsymbol{E}_{\text{hom ref}}$	0	$[0, \frac{1}{2})$	homozygous ref
$\boldsymbol{E}_{\text{ADO to ref}}$	0	$[\frac{1}{2}, 1)$	heterozygous
$\boldsymbol{E}_{\text{err ref}}$	$\{0, \frac{1}{2}\}$	1	homozygous alt
$\boldsymbol{E}_{\text{err alt}}$	$\{\frac{1}{2}, 1\}$	0	homozygous ref
$\boldsymbol{E}_{\text{het}}$	$\frac{1}{2}$	$(0, 1)$	heterozygous
$\boldsymbol{E}_{\text{ADO to alt}}$	1	$(0, \frac{1}{2}]$	heterozygous
$\boldsymbol{E}_{\text{hom alt}}$	1	$(\frac{1}{2}, 1]$	homozygous alt

ADO – allele dropout, alt – alternative, err – error, het – heterozygous,
hom – homozygous, ref – reference

1.5 Bulk background: Accounting for Amplification Errors and Allele Dropout

1.5.1 Defining the Event Space

Using the above model for a genome position g, we can compute the likelihood of any combination of possible underlying alternative nucleotide frequencies in the single cell ($\tilde{\theta}_s$) and the bulk sequencing sample ($\tilde{\theta}_b$), given the respective data. All the possible combinations of allele frequencies in the two samples define a two-dimensional space of allele frequency likelihood density estimates $\boldsymbol{E}$ (Equation 4, Supplementary Figure 2, Supplementary Supplementary Table 2). We then define mutually exclusive sub-spaces of $\boldsymbol{E}$ as single cell events, for example the dropout of an alternative allele across allele frequencies $\boldsymbol{E}_{\text{ADO to ref}} = \{0\}_{\tilde{\theta}_s} \times [\frac{1}{2}, 1)_{\tilde{\theta}_b}$, providing a set of events $\mathfrak{E}$. For these definitions, we always assume that—regarding a particular genomic site—the bulk cell

population can only consist of a maximum of two sub-populations that are exactly one mutated allele apart from each other (Supplementary Figure 2, lower panel). At allele frequencies $\theta_b \in \{0, 0.5, 1\}$, we assume a homogeneous population of only homozygous reference, heterozygous, or homozygous alternative cells, respectively. At frequencies in between, we assume a mixture of heterozygous cells with homozygous reference cells ($\theta_b \in (0, 0.5)$), or with homozygous alternative cells ($\theta_b \in (0.5, 1)$). For the above example ($\boldsymbol{E}_{\text{ADO to ref}} = \{0\}_{\tilde{\theta}_s} \times [\frac{1}{2}, 1)_{\tilde{\theta}_b}$), this means that we can resolve a contradiction between the likelihood estimate at $\tilde{\theta}_s = 0$, which indicates a homozygous reference single cell, and the likelihood density estimate across $\tilde{\theta}_b \in [0.5, 1)$, which indicates that the bulk does not contain any homozygous reference cells. Based on our assumption, we decide to trust the bulk sample over the single cell sample, meaning that the bulk is a mixture of a maximum of two sub-populations, and can only contain heterozygous and homozygous alternative cells, rendering a homozygous reference single cell impossible and thus the result of an allele dropout.

To evaluate whether this assumption of only two sub-populations in the bulk is safe to make, we can examine all the possible ways in which this assumption may be violated. This requires that we have a sequence of two mutational events affecting the same genomic site, each generating a new sub-population. To know which types of mutational event sequences to consider, we note that the same point mutation has been observed to occur independently in different single cells from the same individual [21], possibly due to selection for an advantageous effect of that mutation. However, the net effect of such parallel evolution still restricts the cell bulk to two sub-populations with distinct genotypes[1]. At the same time, we are not aware of cases where different cells from the same population acquire different point mutations at the exact same locus, and expect this to be highly unlikely. Instead, a third sub-population will usually be generated by

[1]As we are looking at different single cells separately, this also means that we do not have to worry about this kind of violation of the infinite sites assumption. Such parallel evolution only becomes problematic for phylogenetic reconstruction approaches that assume infinite sites, which would assume that each mutation only happens once in the tree.

a loss of heterozygosity event on a larger scale, covering a mutated site [21, 42].

Thus, if we start with a homozygous reference bulk, a first event would introduce an alternative allele mutation and a second event in that sub-population would lead to the loss of either the reference allele or the alternative allele. Losing the recently generated alternative allele leads to an allele frequency in that sub-population that is similar to the homozygous reference genotype (it only has coverage of the reference allele), and this sub-population is thus indistinguishable from the original homozygous reference population. ProSolo will correctly identify any single cells with the loss event as homozygous reference (Supplementary Figure 2).

However, losing the reference allele leads to a sub-population that only has coverage of the alternative allele and is thus similar to a homozygous alternative genotype and clearly distinguishable from the other two genotypes. While this type of event was not observed in a systematic survey of multiple mutational hits per site [21], it has coincidentally been observed in a recent study that explicitly modeled copy number alterations in single cells for their phylogenetic reconstruction of cell lineages (mutation LINGO2:2 in Satas et al. [42]). Thus, we expect this type of event to be rare, but possible. In this case, as the alternative allele was only introduced in a sub-population and the loss of the reference allele is only present in an even smaller sub-population of that, the bulk frequency of this alternative allele will be well below 0.5. Here, the ProSolo model assumes the homozygous alternative genotype of this sub-population impossible and instead calls cells from that sub-population as heterozygous (Supplementary Figure 2). However, this only concerns the genotype, while the presence of the mutation will be identified correctly. As MonoVar is the only other variant calling software that can even genotype beyond calling the presence or absence of a mutation, this could only be considered a drawback compared to that tool.

As germline homozygous alternative sites are very rare compared to germline homozygous reference sites (due to the reference genome being biased towards more common

alleles), we expect the symmetric case[m] to be much less likely to be observed.

Finally, starting from a heterozygous germline genotype in the bulk will be more likely than starting from a homozygous alternative genotype, but a lot less likely than starting from a homozygous reference genotype. From this starting point, three sub-populations regarding the same alternative allele can only be generated by two events in opposite directions in distinct cells from that population: one point mutation or loss event towards the reference and one point mutation or loss event towards the alternative allele, with the loss events much more likely to affect any site than the point mutations. Whether the resulting bulk alternative allele frequency then ends up above or below 0.5, depends on which event happens earlier and/or grows faster within the population. As a result, the homozygous genotype (reference or alternative) with the lower frequency will erroneously be considered a heterozygous single cell genotype, but ProSolo will correctly call the more frequent (and thus probably more relevant) homozygous genotype. However, we are not aware of any such events being reported in the literature and expect them to be vary rare.

We conclude that the assumption is reasonable to make and we only expect it to lead to the misclassification of homozygous alternative single cell sites as heterozygous when they have been generated by the introduction of an alternative allele via a point mutation, followed by an allelic loss of the reference allele in some daughter cells. We also discuss possible respective cases in the context of the tumor single cells from the data of Wang et al. [48] (Supplement Section 2.5).

It should be possible to mitigate such cases with further extensions of the model to account for copy number changes. As in Satas et al. [42], this would require separately obtaining copy number profiles that are then provided as input to ProSolo, but we do not yet see a standardized way of obtaining such profiles from single cell DNA-sequencing data. So far, such copy number profiles seem to have usually been obtained with custom

[m] A mutation towards the reference with a subsequent complete loss of the originally homozygous alternative allele.

scripts tailored to specific datasets (for example Baslan et al. [2], Satas et al. [42], Kuipers et al. [22]), but when more generalized approaches for obtaining such profiles become available, they could then be used to adjust the exact model setup for each site based on the local copy number profile. Newer versions of varlociraptor, whose variant calling functionality ProSolo uses in the background, already allow for a chromosome-specific specification of copy numbers[n]. Thus, it should be possible to extend this to site-specific copy numbers in the future and to extend the ProSolo model to arbitrary ploidies.

1.5.2 Posterior Probabilities of Events

Further assuming that there is no informative prior information about different allele frequencies (i.e. assuming a flat prior across all possible allele frequencies in both samples), we can then calculate the likelihood of such an event as the product of the likelihoods of the allele frequency ranges in the two samples. For $\boldsymbol{E}_{\text{ADO to ref}}$—and using Equation S 24 to calculate the likelihoods of the allele frequency ranges—this for example becomes:

$$
\begin{aligned}
P(\boldsymbol{Z}^s, \boldsymbol{Z}^b \mid \boldsymbol{E}_{\text{ADO to ref}}) &= P(\boldsymbol{Z}^s \mid \theta_s = 0) \times P(\boldsymbol{Z}^b \mid \theta_b \in [\frac{1}{2}, 1)) \\
&= \sum_{k=0}^{l} \left\{ P(\boldsymbol{Z}^s \mid \rho_s = \frac{k}{l}) \times \mathfrak{B}\mathfrak{B}_{l,\alpha,\beta}(\rho_s = \frac{k}{l}) \right\} \times \\
&\quad \sum_{m=\frac{n}{2}}^{n-1} P(\boldsymbol{Z}^b \mid \theta_b = \frac{m}{n})
\end{aligned}
\tag{S 25}
$$

Here, we use the fact that for the bulk sample without amplification, we can assume that $\theta_b = \rho_b$ and thus use a simpler form of Equation S 23. Also, we take n to be the total number of bulk reads and m to be the possible numbers of reads with the alternative allele for this event—analogous to k and l for the single cell sample, and amounting to only taking point estimates of the likelihoods at possible alternative allele counts given

[n]See the "ploidy" definition in this documentation section: `https://varlociraptor.github.io/docs/calling/#configuring-the-joint-prior-distribution`

the total coverage of the examined genomic site.

As all the defined events (Equation 5) are mutually exclusive (Supplementary Figure 2, Supplementary Table 2), the sum of the likelihoods of all events yields the marginal probability:

$$
\begin{aligned}
P(\boldsymbol{Z}^s, \boldsymbol{Z}^b) = P(\boldsymbol{Z}^s, \boldsymbol{Z}^b \mid \boldsymbol{E}) = \sum_{\boldsymbol{E}_e \in \mathfrak{E}} & P(\boldsymbol{Z}^s, \boldsymbol{Z}^b \mid \boldsymbol{E}_e) \\
= & P(\boldsymbol{Z}^s, \boldsymbol{Z}^b \mid \boldsymbol{E}_{\text{hom ref}}) \ + \ P(\boldsymbol{Z}^s, \boldsymbol{Z}^b \mid \boldsymbol{E}_{\text{hom alt}}) + \\
& P(\boldsymbol{Z}^s, \boldsymbol{Z}^b \mid \boldsymbol{E}_{\text{het}}) + \\
& P(\boldsymbol{Z}^s, \boldsymbol{Z}^b \mid \boldsymbol{E}_{\text{err alt}}) \ + \ P(\boldsymbol{Z}^s, \boldsymbol{Z}^b \mid \boldsymbol{E}_{\text{ADO to alt}}) + \\
& P(\boldsymbol{Z}^s, \boldsymbol{Z}^b \mid \boldsymbol{E}_{\text{err ref}}) \ + \ P(\boldsymbol{Z}^s, \boldsymbol{Z}^b \mid \boldsymbol{E}_{\text{ADO to ref}})
\end{aligned}
\tag{S 26}
$$

Then, using Equation S 22 for the likelihood of ρ_s in the single cell sample and the equivalent relationship for the likelihood of θ_b in the bulk sample (with j as an index running through all bulk sample reads), we get:

$$
\begin{aligned}
P(\boldsymbol{Z}^s, \boldsymbol{Z}^b \mid \boldsymbol{E}_{\text{ADO to ref}}) = & \sum_{k=0}^{l} \left\{ \prod_{i=1}^{l} P(Z_i^s \mid \rho_s = \frac{k}{l}) \times \mathfrak{B}\mathfrak{B}_{l,\alpha,\beta}(\rho_s = \frac{k}{l}) \right\} \times \\
& \sum_{m=\frac{n}{2}}^{n-1} \prod_{j=1}^{n} P(Z_j^b \mid \theta_b = \frac{m}{n})
\end{aligned}
\tag{S 27}
$$

Using Equation S 21 for the likelihood of an individual read in the single cell sample given a ρ_s and the respective formulation for an individual read in the bulk sample given a θ_b, this will calculate the likelihood of the `ADO to ref` event as:

$$
\begin{aligned}
&P(\boldsymbol{Z}^s, \boldsymbol{Z}^b \mid \boldsymbol{E}_{\text{ADO to ref}}) = \\
&\quad \sum_{k=0}^{l} \left\{ \prod_{i=1}^{l} \left((1-\pi_i^s) + a^s(Z_i^s) \times \pi_i^s \times (1-\frac{k}{l}) + p^s(Z_i^s) \times \pi_i^s \times \frac{k}{l} \right) \times \right. \\
&\qquad\qquad \left. \mathfrak{BB}_{l,\alpha,\beta}(\rho_s = \frac{k}{l}) \right\} \times \\
&\quad \sum_{m=\frac{n}{2}}^{n-1} \left\{ \prod_{j=1}^{n} \left((1-\pi_j^b) + a^b(Z_j^b) \times \pi_j^b \times (1-\frac{m}{n}) + p^b(Z_j^b) \times \pi_j^b \times \frac{m}{n} \right) \right\}
\end{aligned} \tag{S 28}
$$

Analogously, the likelihoods for all of the events as specified in Supplementary Figure 2 can be computed and their sum according to Equation S 26 is the marginal probability. We can use the marginal probability to calculate the posterior probability of the `ADO to ref` event with:

$$
\begin{aligned}
&P(\boldsymbol{E}_{\text{ADO to ref}} \mid \boldsymbol{Z}^s, \boldsymbol{Z}^b) = \\
&\quad \frac{1}{\sum_{\boldsymbol{E}_e \in \mathfrak{E}} P(\boldsymbol{Z}^s, \boldsymbol{Z}^b \mid \boldsymbol{E}_e)} \times \\
&\quad \sum_{k=0}^{l} \left\{ \prod_{i=1}^{l} \left((1-\pi_i^s) + a^s(Z_i^s) \times \pi_i^s \times (1-\frac{k}{l}) + p^s(Z_i^s) \times \pi_i^s \times \frac{k}{l} \right) \times \right. \\
&\qquad\qquad \left. \mathfrak{BB}_{l,\alpha,\beta}(\rho_s = \frac{k}{l}) \right\} \times \\
&\quad \sum_{m=\frac{n}{2}}^{n-1} \left\{ \prod_{j=1}^{n} \left((1-\pi_j^b) + a^b(Z_j^b) \times \pi_j^b \times (1-\frac{m}{n}) + p^b(Z_j^b) \times \pi_j^b \times \frac{m}{n} \right) \right\}
\end{aligned} \tag{S 29}
$$

With analogous equations, we can compute the posterior probability for all of the events defined by ProSolo (Supplementary Figure 2, Supplementary Table 2). Each of them is reported in the output VCF or BCF file. Based on the implicit single cell genotypes as given in Supplementary Table 2, we can then compute posterior probabilities for genotypes in the single cell by summing the posterior probabilities of all the events

that imply each genotype. For the heterozygous case (light blue fields in Supplementary Figure 2), this would be:

$$\begin{aligned} P(\text{heterozygous}_s \mid \boldsymbol{Z}^s, \boldsymbol{Z}^b) = \; & P(\boldsymbol{E}_{\text{ADO to ref}} \mid \boldsymbol{Z}^s, \boldsymbol{Z}^b) \; + \\ & P(\boldsymbol{E}_{\text{het}} \mid \boldsymbol{Z}^s, \boldsymbol{Z}^b) \; + \\ & P(\boldsymbol{E}_{\text{ADO to alt}} \mid \boldsymbol{Z}^s, \boldsymbol{Z}^b) \end{aligned} \tag{S 30}$$

The genotype with the maximum probability among the three possible genotypes is then chosen. Similarly, we can also compute posterior probabilities for any compound event. For example, the overall posterior probability of an allele dropout at a particular site (`ADO` events in Supplementary Figure 2 and Supplementary Table 2) is:

$$\begin{aligned} P(\text{allele dropout} \mid \boldsymbol{Z}^s, \boldsymbol{Z}^b) = \; & P(\boldsymbol{E}_{\text{ADO to ref}} \mid \boldsymbol{Z}^s, \boldsymbol{Z}^b) \; + \\ & P(\boldsymbol{E}_{\text{ADO to alt}} \mid \boldsymbol{Z}^s, \boldsymbol{Z}^b) \end{aligned} \tag{S 31}$$

Another example is the probability of the presence of any alternative allele at a site (all blue fields in Supplementary Figure 2, Equation 10 in the main manuscript). This posterior probability is the one used for the main benchmarking of ProSolo against the other existing single cell specific variant callers (MonoVar, SCAN-SNV, SCcaller, and SCIPhI), as SCcaller and SCIPhI only call the presence (vs. absence) of an alternative nucleotide and this is thus the only measure we can compare all tools on.

1.6 Implementation

ProSolo is an easy-to-use command-line tool (following usability standards [46]) and its source code is available at `https://github.com/prosolo/prosolo`, including instructions for an easy installation (via Bioconda [13]). The main contribution in terms of software that ProSolo's development provided, is the implementation of its comprehen-

sive statistical model that jointly looks at a single-cell and a bulk sample. This was implemented as a part of Varlociraptor, a variant calling library implemented in Rust [23]. Varlociraptor already makes use of all available sources of information regarding biases of normal sequencing data, induced by ambiguous alignments and sequencing errors, but had to be extended to accommodate this type of sample combination and to allow for accounting for amplification bias. Because Varlociraptor had originally been specializing in indels and structural variants, implementing ProSolo also meant further investment in expanding Varlociraptor's capabilities for basic SNV analysis and required some improvements in the handling of sequencing alignment data in the upstream library rust-htslib [41], a Rust wrapper around htslib [16]. Further, we optimized Varlociraptor's calculations wherever possible, for example by reducing the number of redundant likelihood calculations and by caching frequently reused values. And finally, numerical issues required an addition to the upstream library rust-bio [19].

False Discovery Rate (FDR) Control of Event Probabilities Last—but not least—ProSolo can control the false discovery rate of any set of single cell events that a user defines. At any scale of high throughput sequencing data (from targeted panels over whole exome sequencing to whole genome sequencing), we generate a large amount of event calls and thus have to control for false discoveries, e.g. by controlling the false discovery rate [3]. Using Varlociraptor's functionality [23], we can estimate thresholds on our posterior probabilities based on the approach described by Müller et al. [31, 32]. For ProSolo, we extended Varlociraptor's capabilities from estimating such thresholds for the posterior probability of a single event to estimating thresholds for any combination of the above defined events. For example, to control the false discovery rate for allele dropout events, we jointly obtain a threshold for the combination of the two ADO events in Figure 3D. Or, to control the false discovery rate in identifying the presence of an alternative allele, we jointly obtain a threshold for the combination of all the

respective events in Equation 10. This functionality, like the improvements mentioned in Section 1.6, is now also available in Varlociraptor's subcommands.

2 Supplementary Note 2: Benchmarking

All code used for benchmarking is available at: `https://www.github.com/prosolo/benchmarking_prosolo`.

2.1 Datasets and Ground Truths

We have performed our benchmarking analyses on two datasets, one previously published for the evaluation of SCcaller by Dong et al. [11] and one generated especially for this Benchmarking (see Figure 4 in the main manuscript). For them, we generated different kinds of ground truths:

Whole genome sequencing of almost identical kin-cells from a cell-line [11]. The first dataset is from the publication of a previous variant caller for single cell sequencing data generated using MDA, SCcaller (Dong et al. [11]; Figure 4a). They started with one initial cell from a cell line and grew it in several steps (a complete overview of their setup is given in Figure 1 of Dong et al. [11]). After an initial mini-expansion, they selected one single cell as the founder for their secondary IL expansion into 20-30 cells. From this they extracted two single cells (IL-11 and IL-12) that were sequenced after multiple displacement amplification (MDA). Both cells have a minimum coverage of five of at least 80% of genomic sites (with only about 90% of the used reference genome represented, as this includes a largely uncovered chromosome Y and alternative haplotype contigs which would only be covered if the particular haplotype is present), with IL-11 having a more even coverage that leads to more sites with a usable coverage (Supplementary Figure 3A). The rest of the kindred cells from that IL clone was

then used as a bulk sequencing sample without MDA (IL-1C), showing a coverage profile similar to the whole genome amplified single cells (Supplementary Figure 3A). We generated the ground truth genotypes for this dataset from the kindred clone IL-1C, which should be only very few cell divisions away from the single cells, and thus have almost no difference in the somatic mutations acquired. The ground truth genotype was generated separately for variant sites and homozygous reference sites: for variant sites, GATK HaplotypeCaller was run, followed by variant quality score recalibration; for homozygous reference sites, we used bcftools mpileup to identify sites with a coverage above 25 but no alternative nucleotide present. This bulk sample (IL-1C) was only used as a ground truth and was not provided as input to any of the software compared here. Three more distant clones (C1, C2, C3 in Figure 4a), grown from the cells after the initial mini-expansion, were jointly used as a further bulk sample for SCcaller and ProSolo (see Software and Parameters below). With three separate bulk sequencing runs, their joint coverage of about 50 for 80% of genomic sites is much higher than for the MDA single cells and the unamplified IL-1C bulk (Clones in Supplementary Figure 3A).

Whole exome sequencing of five human granulocytes with a pedigree ground truth. For the second benchmarking dataset, Blood was taken from a patient after informed consent and granulocytes were sorted out in several steps: Initially, CD3- cells were captured via Magnetic-Activated Cell Sorting (MACS); among them, CD66b+ granulocytes were filtered out via Fluorescence Activated Cell Sorting (FACS) (Figures 4b and 4). Single cells from that cell population were isolated using a Fluidigm C1 microfluidics chip and subjected to multiple displacement amplification (MDA) using the GE Healthcare GenomiPhi Kit in its version adapted to that microfluidic chip. With a panel of 16 custom primer pairs covering different genes across chromosomes (Supplementary Table 3) used in a quantitative real-time PCR, we selected granulocytes where

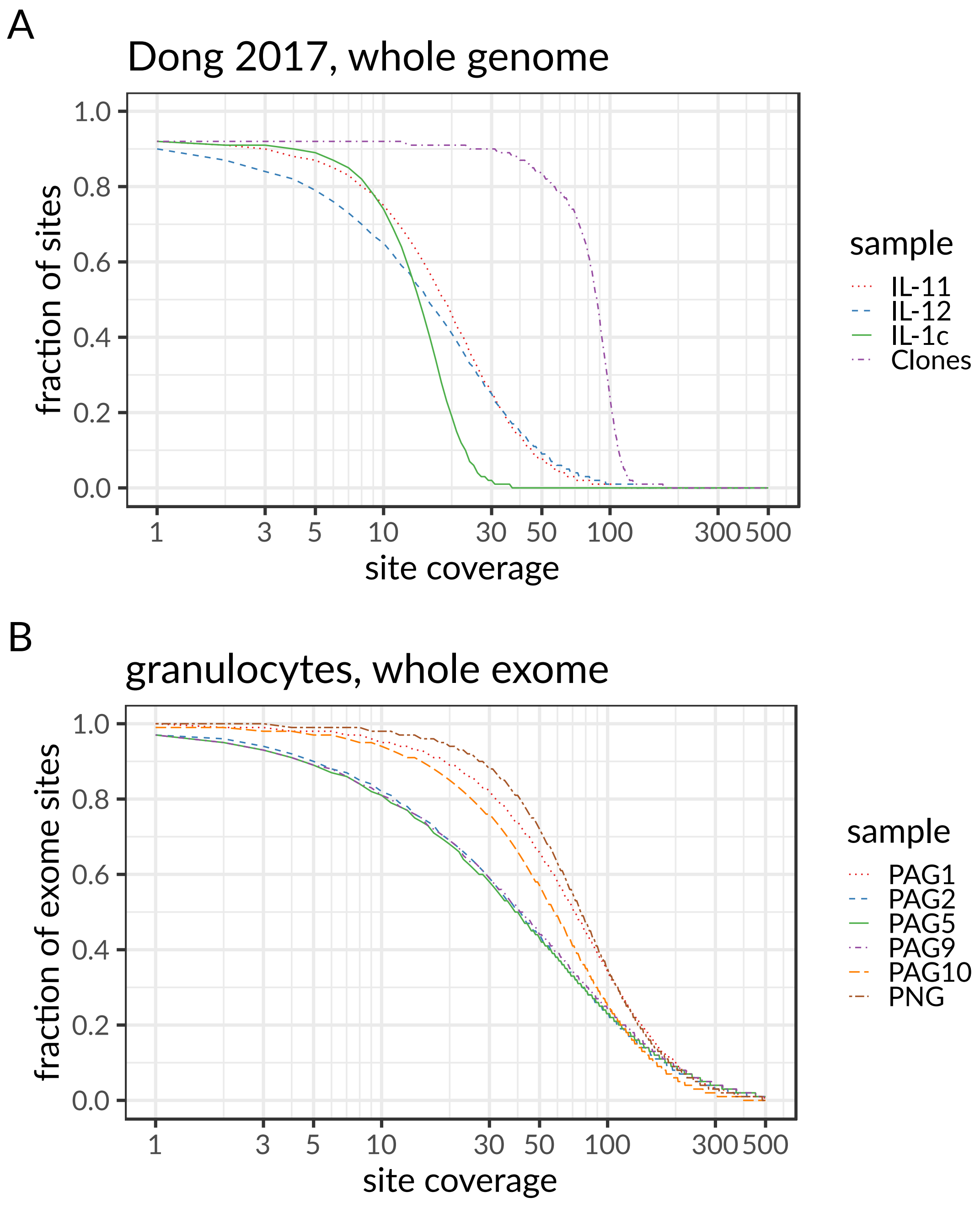

Supplementary Figure 3: Sequencing coverage of targeted genomic sites for multiple displacement amplified (MDA) single cells and respective bulk samples (continued on following page). Panel **A** shows the coverage of the single cells (IL-11, IL-12), the kindred ground truth clone (IL-1c) and the more distant background bulk (Clones C1, C2, and C3 joined into one sample) of the dataset from Dong et al. [11]. Panel **B** shows the coverage of the single cells (PAG1, PAG2, PAG5, PAG9, PAG10) and the background bulk (PNG) of the granulocyte data generated for this evaluation.

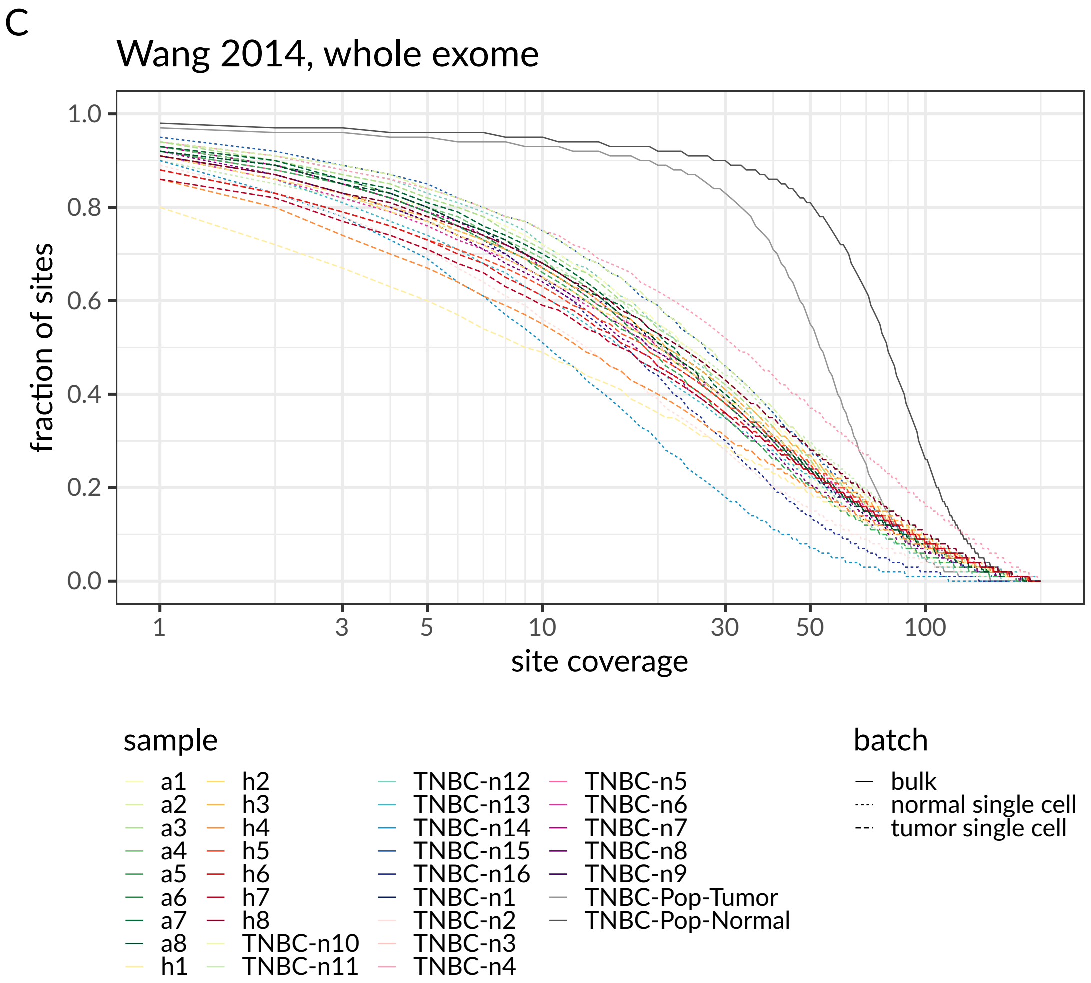

Supplementary Figure 3: Sequencing coverage of targeted genomic sites for multiple displacement amplified (MDA) single cells and respective bulk samples (continued from previous page). Panel **C** shows the coverage of the single cells (a1 to a8, h1 to h8, and TNBC-n1 to TNBC-n16), and the bulk samples (TNBC-Pop-Tumor and TNBC-Pop-Normal) that were used as ground truth for the normal single cells (with validated somatic variants added) and as ground truth for the tumor single cells (with validated somatic variants removed), respectively Wang et al. [48].

Supplementary Table 3: Primers used for selection of well amplified single cell samples via quantitative real-time PCR. These were taken from Table S3 in Zong et al. [56].

chromosome	forward primer	reverse primer
chr1	AGGAAAGGCATACTGGAGGGACAT	TTAGGGATGGCACCACACTCTTGA
chr2	TCCCAGAGAAGCATCCTCCATGTT	CACCACACTGCCTCAAATGTTGCT
chr3	TCAAGTTGCCAGCTGTGGCTGTAT	AGAAGGGCATTTCCTGTCAGTGGA
chr4	ATGGGCAAATCCAGAAGAGTCCAG	CCATTCACTTCCTTGGAAAGGTAGCC
chr5	AATAGCGTGCAGTTCTGGGTAGCA	TTCACATCCTGGGAGGAACAGCAT
chr6	TGAATGCCAGGGTGAGACCTTTGA	TGTTCATTATCCCACGCCAGGACT
chr7	ACCAAAGGAAAGCCAGCCAGTCTA	ACTCCACAGCTCCCAAGCATACAA
chr9	TCCCAGCTCTCTCTCTTGCATCTT	AGTGAAGCTGGTGTATGCAGAGGT
chr12	AGAGGGCTGCTTTATGCAGGTG	CTACATTTGGGTCTTTGCTGCCATG
chr13	AGCAGCCCCAGGCAGAT	CGGAGAGGACGGTCACGTTTAC
chr14	CGGAGAGGACGGTCACGTTTAC	CGTGGGAGTTTTGAAATGCGATGT
chr15	CCTGTCTCTGCTCCTGCG	TGCACACATGCACAGTGGAG
chr16	CTCCAAGGTTCTGCAGCCTC	GGTATGACTACACATTCAGGCTGG
chr17	GTGGTACATAGTGCATGGTCCG	GGCGACATACCCCAACTTCATAAG
chr18	CGTTCTTAGGACCAAAGGGCTG	CCAGCATCCATGTCTCTGCAC
chr19	GCCCAGAGCGCCTGA	CCAGCCCCTGGACCACT

at least 15 of these loci were properly amplified. For those cells, we performed whole exome capture using the Agilent SureSelectXT Human All Exon V5+UTRs Kit. The paired-end sequencing library (2x101bp) was then prepared with the TruSeq V3 Kit and sequenced on a HiSeq 2500. From the remaining sorted cell population, we extracted bulk DNA and submitted it to the same whole exome capture and DNA sequencing without MDA.

For the ground truth of this dataset, we could leverage previously published bulk whole exome sequencing data from the same person, their parents and three siblings (Hoell et al. [14]; Figure 4b). For ground truth alternative allele calls, we ran three pedigree-aware variant callers on the family whole exome data. To be able to run them

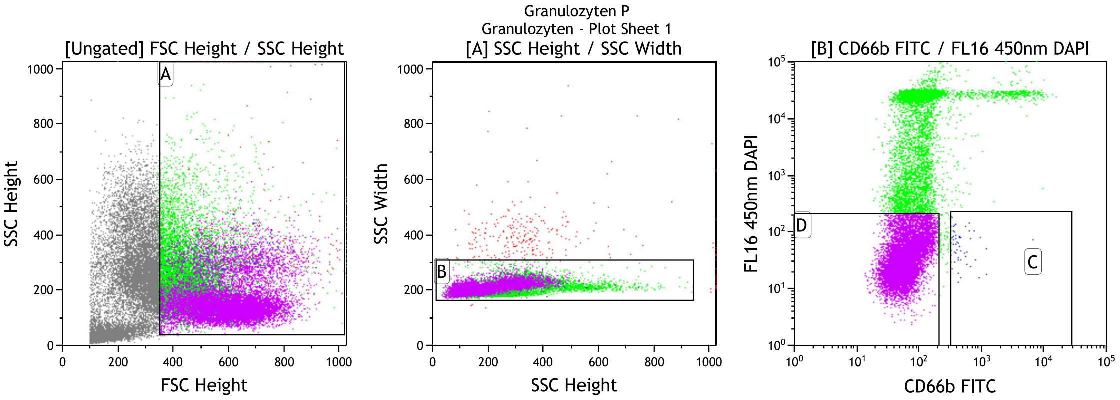

Granulozyten P 11.08.14

Gate	Number	%Total	%Gated	Logic
All	35.779	100,00	100,00	Ungated
A	22.423	62,67	62,67	A
B	22.160	61,94	98,83	B AND A
C	59	0,16	0,27	C AND B AND A
D	11.813	33,02	53,31	D AND B AND A

Supplementary Figure 4: 59 CD66b+ granulocytes were sorted out from a total population of 35779 CD3-T cell depleted peripheral blood cells (gate C in the right-most panel) and from these, five single cells were chosen where whole genome amplification had yielded DNA for at least 15 out of 16 control loci.

reproducibly, we made all three of them available via bioconda [13]:

- BEAGLE 4.0 [6], bioconda version: `beagle=4.0_06Jun17=1`
- polymutt [24], bioconda version: `polymutt=0.18=0`
- FamSeq in MCMC (`-method 3`) mode [37, 38]), bioconda version: `famseq=1.0.3=0`

From their calls, we created a consensus by only including sites where either all callers agree on a genotype or where a maximum of one caller has a *missing* (as opposed to different genotype) call, while the other two callers agree on the genotype (Supplementary Figure 5). The use of the full pedigree ensures high confidence genotypes—however, please bear in mind that these represent the germline genotype. Single cells will have accumulated somatic genotypes that differ from the germline and we are bound to misinterpret those as false positive calls in our ground truth comparison. At the same time, the number of such somatic variants will be small compared to the number of germline

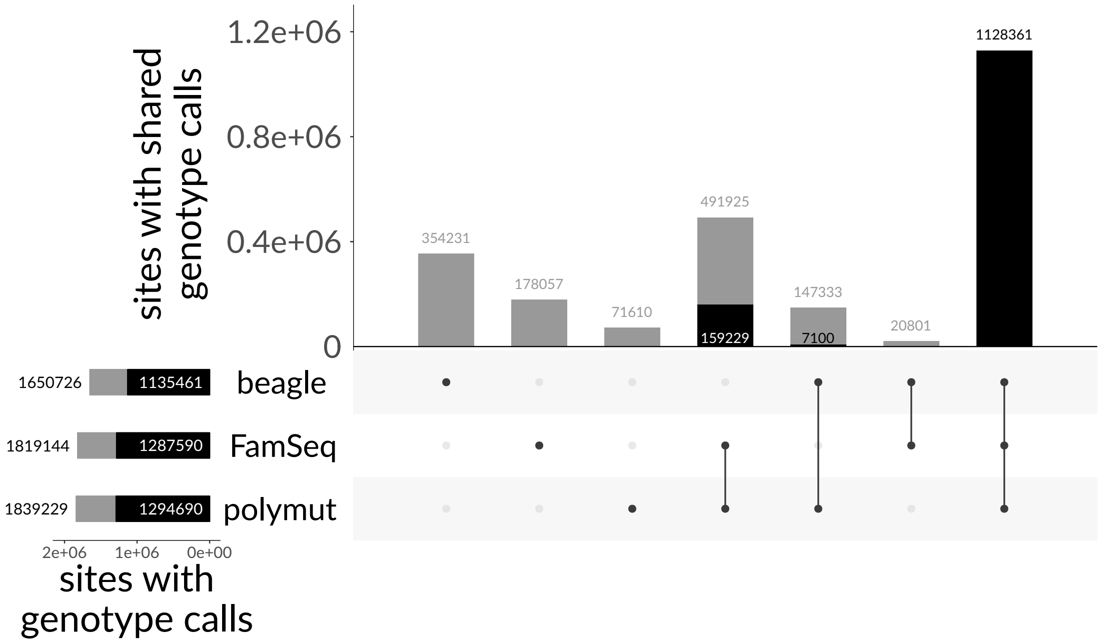

Supplementary Figure 5: Constructing the germline ground truth genotype for our granulocytes dataset from the consensus of three pedigree-aware callers. Variants were called on a previously generated whole exome bulk sequencing data set of the person's family (Figure 4), which is separate from the single cell and bulk whole exome sequencing data generated for this study. Variants were called using beagle, FamSeq and polymutt with total counts of individual tools (bottom left bar plot) and tool intersections of sites called (top bar plot) in grey. Black fractions of bars represent the sites selected for our consensus: sites where either all callers agree upon a genotype or at least two callers agree upon a particular genotype and the third tool has a missing genotype (i.e. doesn't contradict the genotype called by the other two).

variants, and should hence keep our overall ground truth comparison valid.

Single nucleus exome sequencing of 16 normal and 16 tumor cells from a triple negative breast cancer (TNBC) [48]. The third dataset is a published dataset from a TNBC patient, with both tumor and matched normal cells available. For each, 16 cells and a bulk sample were whole exome sequenced (Figure 4c). For the 32 single cells, library preparation was performed on isolated single nuclei [48]. In addition, Wang et al. [48] provide a list of 373 non-synonymous clonal and 94 non-synonymous

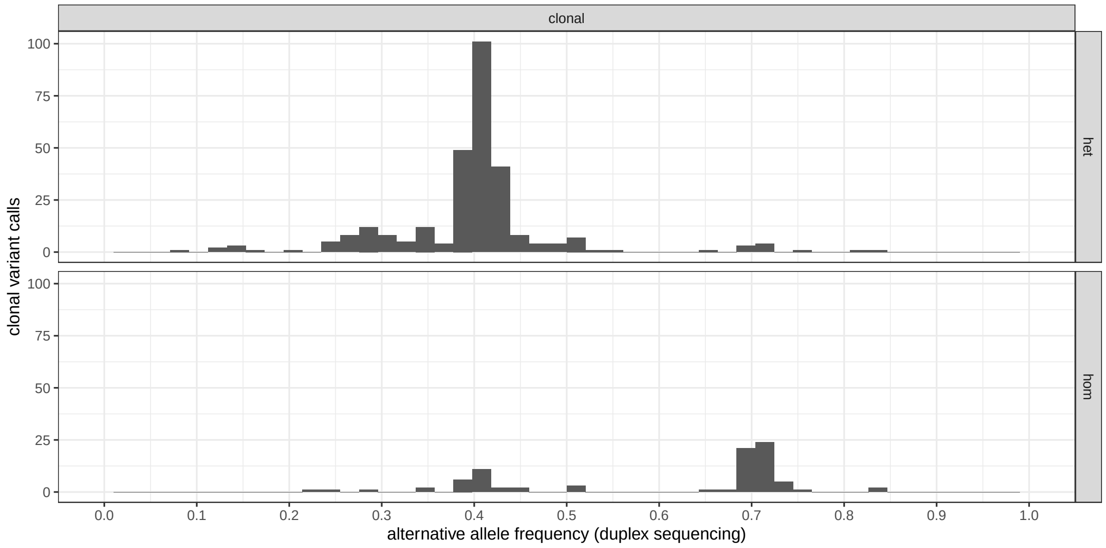

Supplementary Figure 6: The number of clonal variants with a respective alternative allele frequency, stratified by the genotype (hom for homozygous alternative and het for heterozygous) provided by Wang et al. [48] for their somatic ground truth list. We consider the peak around 0.4 to represent genuine heterozygous variants and the peak around 0.7 to represent genuine homozygous alternative variants, with a tumor sample purity in the duplex sequencing experiment of somewhere around those 0.7.

subclonal somatic variants (Supplementary Tables 6 and 7 in Wang et al. [48]) whose presence they validated with deep targeted duplex sequencing. As they provide a duplex sequencing based alternative allele frequency, we examined the alternative allele frequency distribution of the non-synonymous clonal variants, stratified by the specified zygosity (Supplementary Figure 6). This clearly shows two alternative allele frequency peaks, one at around 0.4 and the other at roughly double the alternative allele frequency around 0.7. These two peaks should correspond to heterozygous (0.4) and homozygous alternative variants (0.7), respectively. As these are supposed to be clonal variants in the tumor, the remaining fraction of 0.3 beyond the homozygous alternative variants peak (from 0.7 to 1) should be contamination with normal cells (without these somatic variants). This indicates a duplex sample tumor purity of around 0.7. Unexpectedly, we also see the 0.7 peak in variants that Wang et al. [48] classified as heterozygous and the 0.4 peak in variants that Wang et al. [48] classified as homozygous alternative (Supplementary Figure 6). It is unclear from Wang et al. [48] and its supplementary material how the original variant classifications were generated, we assume that this is based on the genotypes obtained for the single cells. But as the single cells were genotyped with standard variant calling software (GATK UnifiedGenotyper), they very likely suffer from considerable allelic dropout and we thus reclassified the clonal variants' genotypes based on the duplex sequencing. If the alternative allele frequency determined by the ultra-deep duplex sequencing was below 0.6, we classified the variant as heterozygous, and as homozygous alternative if this frequency was above (or equal to) 0.6 (Supplementary Figure 6). Then, depending on the single cell, we used two different ground truths for two separate evaluations:

1. For the tumor single cells, we constructed a ground truth from the normal bulk sample (as the tumor bulk sample was used for calling with ProSolo) using the same methodology as used for the cell line data (GATK HaplotypeCaller for variant sites, bcftools mpileup for non-variant sites). Please bear in mind that this ground

truth will mostly only contain germline variants and only few somatic mutations that appeared early enough in the patient's life to rise to high somatic frequencies. Thus, similar caveats as for the germline ground truth in the granulocyte dataset apply. To mitigate this to a certain degree, we added the 373 clonal validated somatic variants, as these are expected to be present in all tumor single cells.

2. For the normal single cells, we constructed a ground truth from the tumor bulk sample (as the normal bulk sample was used for calling with ProSolo) using the same methodology as used for the cell line data (GATK HaplotypeCaller for variant sites, bcftools mpileup for non-variant sites). To reduce the presence of false-positive somatic variants in this ground truth, we removed all 467 validated somatic variants (clonal and subclonal) from the ground truth.

Further, we determined the of the validated somatic variants for all software run on this dataset.

2.2 Software and Parameters

ProSolo was compared against the available tools for single nucleotide variant calling in single cell sequencing data from multiple displacement amplified (MDA) DNA, MonoVar [55], SCAN-SNV [29], SCcaller [11], and SCIPhI [44].

For ProSolo (version `0.6.1` installed via bioconda), each single cell was called against the respective cell's bulk (the joined clones 1, 2 and 3 for the [11] dataset, PNG for our own dataset, see Figure 4). Candidate sites to call were generated using bcftools mpileup, taking all sites where at least one read covered a non-reference nucleotide in the single cell or its respective bulk. We ran two different modes of the software: Calling sites with a minimum read coverage of 1 in the single cell sample (default mode in Figure 1) or including zero-coverage sites, effectively imputing genotypes at these sites from the bulk sample (imputation). The different filter thresholds used represent the

false discovery rate (`--fdr`) that we can control for (Section 1.6).

For MonoVar (version `0.0.1`, which is commit `e0cf2db` cleaned up for the bioconda version we created), parameters were set as recommended in the software repository README [54]. We ran two different modes of the software, with consensus filtering between cells of a population (`-c 1`) and without the filtering (`-c 0`). The different filter thresholds used represent the parameter `-t` being varied. However, turning on the consensus filtering did not cause any substantial changes, possibly due to the low number of cells sequenced (Supplementary Figure 7). Varying the parameter `-t` did not cause any substantial changes in MonoVar results, either.

SCAN-SNV [29] was installed via the install instruction in the repository on 2019-10-30[o] and patched locally to make it run. The pipeline was then run with parameters set as recommended in the software repository `README.md` and the command line help, with the only changes made for a better comparability with the other tools. Namely, we tried to call as sensitively as possible by setting: `--min-sc-alt 1 --min-sc-dp 1 --min-bulk-dp 1`. However, the default pipeline only provides calls for somatic variants (variants only found in single cells, not in the bulk, as opposed to all the present alternative alleles), heavily filtering candidate sites before calling. We thus attempted a fairer alternative allele calling comparison by modifying the pipeline to remove the filter that eliminates alternative alleles also seen in the bulk sample and the filter that eliminates known dbsnp variation[p]. The different filter thresholds used represent the `--fdr` parameter, which—according to the authors—does not formally control the false

[o] This are the install instructions at the repository state at `https://github.com/parklab/scan-snv/blob/62b7dcbc21e6cac64dc384abdfa708120e33819c/README.md`. This includes the following packages and versions from the custom conda channel `https://anaconda.org/jluquette`: `r-scansnv 0.1`, `scansnv 0.9`, `r-fastghquad 1.0` and `natefoo-slurm-drmaa 1.2.0`. Further, it requires manual registration of the java executable for `gatk 3.8`.

[p] I.e. we edited the snakemake `rule scansnv_somatic_sites` to exclude the following requirements: `tab[,bulk.alt] == 0 & tab[,bulk.idx] == '0/0' & tab$dbsnp == '.'`.

discovery rate [29]. For the third dataset, added following a suggestion from peer-review, we had to recreate the conda environment for SCAN-SNV, following newer advice in the issues of the repository[q]. While this allowed us to run the pipeline, the custom rule for ground truth comparison ran into unsolvable version conflicts. After multiple days of debugging attempts, we decided to exclude SCAN-SNV from the analysis of this dataset, also given its performance on the other datasets.

For SCcaller (version `1.2`, as available on bioconda), default parameters were set as recommended in the software repository README [10]. We ran four different modes of the software, varying two things: 1. For the heterozygous candidate sites that SCcaller requires as input, we either used dbSNP [43, 33] entries or generated them from the bulk background sample that was also used for calling in ProSolo, running GATK HaplotypeCaller. As the dbSNP-derived heterozygous candidate sites consistently outperformed those generate from the bulk background sample, only results using dbSNP are reported in the figures, to provide a cleaner comparison. 2. In addition to the recommended settings that require a minimum coverage of 10 (default, `--min 10 --minvar 4 --RD 20`), we also ran SCcaller with reduced coverage requirements to increase sensitivity for a fairer comparison with ProSolo (sensitive, `--min 4 --minvar 1 --RD 10`), thus including sites with a minimum read coverage of 4 and down to one read covering the alternative nucleotide. The three filtering thresholds used represent the filtering levels available for the `-a cutoff` command-line parameter. The filtering threshold gave certain leverage over the precision-recall trade-off and reducing the coverage requirements improved the sensitivity of results (Supplementary Figure 7).

For SCIPhI (version `0.1.4`, as available on bioconda), we ran two different modes: 1. The default parameters of the command line tool (`default`). 2. Fewer iterations

[q]see: `https://github.com/parklab/scan-snv/issues/4#issuecomment-596296645`

(`-l 400000`, because of the excessive run time) and more `sensitive` by turning off all heuristic filters (`--cwm 1 --mnp 1 --ms 1 --bns 0 --bnc 0 --ncf 0 --mnc 1`). On the whole exome dataset with five cells, SCIPhI ran for three weeks for each of these modes. On the whole genome dataset with two cells, an initial run of SCIPhI was killed due to a server problem after running for more than eight weeks. An attempt to restart from intermediate data using the `--il` command line parameter had the model estimations escalating towards unrealistic values, and the crash was thus left unrecoverable. A rerun on a server with more performant CPUs led to the sensitive mode finishing after 5 weeks and the default mode after 7.5 weeks. On the third dataset, added after a suggestion during peer-review, we only ran SCIPhI with default parameters and reduced the iterations to `-l 80000` (and the number of iterations for the mutation to cell assignment to `--ls 40000`) to finish analysis in a reasonable time-frame. No guidance is given for those parameters, but the optimization score seemed to have mostly settled with this amount iterations. With these parameters, this analysis finished within a week for both the tumor cell and the normal cell datasets of Wang et al. [48]. As no option for filtering or thresholding based on sensitivity vs. specificity is available from SCIPhI itself, we parsed output with custom scripts to extract what must be a posterior probability of the presence of a variant at a particular site into a format that ProSolo's false discovery rate control mechanism can handle. This is the value in the last (fourth) FORMAT field, which according to the header annotation is either the GQ or the PL field (five FORMAT fields are specified, but only four values per sample are provided). Judging from the source code[r], this field contains a PHRED scaled posterior probability of the site not being a variant[s]. We parsed this into the probability of a variant being present and passed it into `prosolo control-fdr` to attempt controlling the false discovery rate

[r]For the full relevant source code, see here: `https://github.com/cbg-ethz/SCIPhI/blob/34975f7050f86325f361c58e8146883af4a2d289/src/output.h#L202-L216`

[s]This is 1 minus a value that i is used for determining genotypes by SCIPhI with a fixed cutoff of 0.95 a few lines above: `https://github.com/cbg-ethz/SCIPhI/blob/34975f7050f86325f361c58e8146883af4a2d289/src/output.h#L205`

(Figures 7 and 14).

For scVILP (has no versioning[t]; install instructions had to be amended[u] and manually fixing parsing and dependency issues[v]), we tried two modes:

1. Default parameters as suggested in the README of the software: `https://github.com/mae6/scVILP#identify-the-candidate-loci`.
2. A more sensitive identification of candidates by turning off heuristic filters (`-ms 1 -nmc 1`) during the execution of `loci_identify.py`.

However, scVILP was not able to handle any of our three benchmarking datasets, with the Gurobi solver that it uses always crashing with an out of memory error, even when provided with more than a Tebibyte of RAM. We documented details of runs and crashes for the developers of the tool in an issue in their code repository: `https://github.com/mae6/scVILP/issues/5`.

2.3 Alternative Allele Calling

The most precise single cell variant callers to date, SCcaller and SCIPhI, can only call the presence vs. the absence of an alternative allele (i.e. the heterozygous and the homozygous alternative genotypes called jointly). We have thus focused on this for the main part of our benchmarking.

For ProSolo, the posterior probability of the presence of an alternative allele is calculated with Equation 10, which represent the blue Event areas in Figures 3D and 2.

Calculations of *p*recision and *r*ecall were based on true positives (TP, alternative allele present in the ground truth and called by the respective software), false positives (FP, the respective software calls the presence of an alternative allele that does not exist in

[t]We used the repository at this commit: `https://github.com/mae6/scVILP/tree/ef38bc422d44daafe043c7dcb28112a1f1ee2fe6`

[u]Pull request to amend install instructions: https://github.com/mae6/scVILP/pull/4

[v]Pull request to allow handling of mitochondrial chromosome: `https://github.com/mae6/scVILP/pull/1`; Pull request to fix impossible import of `cdecimal` by upgrading to `decimal` `https://github.com/mae6/scVILP/pull/2`

the ground truth at that site), and false negatives (FN, the respective software makes no call or calls a homozygous reference genotype at a ground truth site with an alternative allele), using the formulas:

$$\begin{aligned} p &= \frac{TP}{TP + FP} \\ r &= \frac{TP}{TP + FN} \end{aligned} \tag{S 32}$$

The whole genome cell line dataset (Figure 4a) seems much less challenging than the other dataset: all methods achieve high precision in alternative allele calling (Figures 7a and 1a), at recall rates of 45% and higher. In comparison with all other tools, ProSolo achieves the most striking increases in recall of nearly 10%. For example, for a precision above .99, its maximum recall is .766 compared to .705 for MonoVar, .687 for SCIPhI and .610 for SCcaller. SCAN-SNV achieves up to .992 precision, but only .0001 recall. This can be explained by it aiming at somatic mutations, while the vast majority of SNVs in a genome will be germline variants[w].

Although a relative increase in recall of about 10% at utmost precision is certainly remarkable, ProSolo demonstrates its power on the second (whole exome) dataset. This dataset is considerably more challenging than the first one. See Figures 1b and 7b for respective results. For this dataset, only SCcaller, SCIPhI and ProSolo achieved a precision above .99, with ProSolo reaching a 20% increase of recall to .178, compared to SCIPhI's .146, and SCcaller with .072 (Figures 1b and 7b). In comparison, MonoVar achieved a maximum precision of only .962. However, this was at a much higher recall (.141) than for example SCcaller (.095 at a precision of .972). SCcaller's decreased recall on this dataset might be due to its estimation of local allelic bias by also taking biases at neighboring sites into account—in whole exome data the number of neighboring sites

[w]E.g. see this comment in the issues of the respective repository: `https://github.com/parklab/scan-snv/issues/5#issuecomment-628107370`

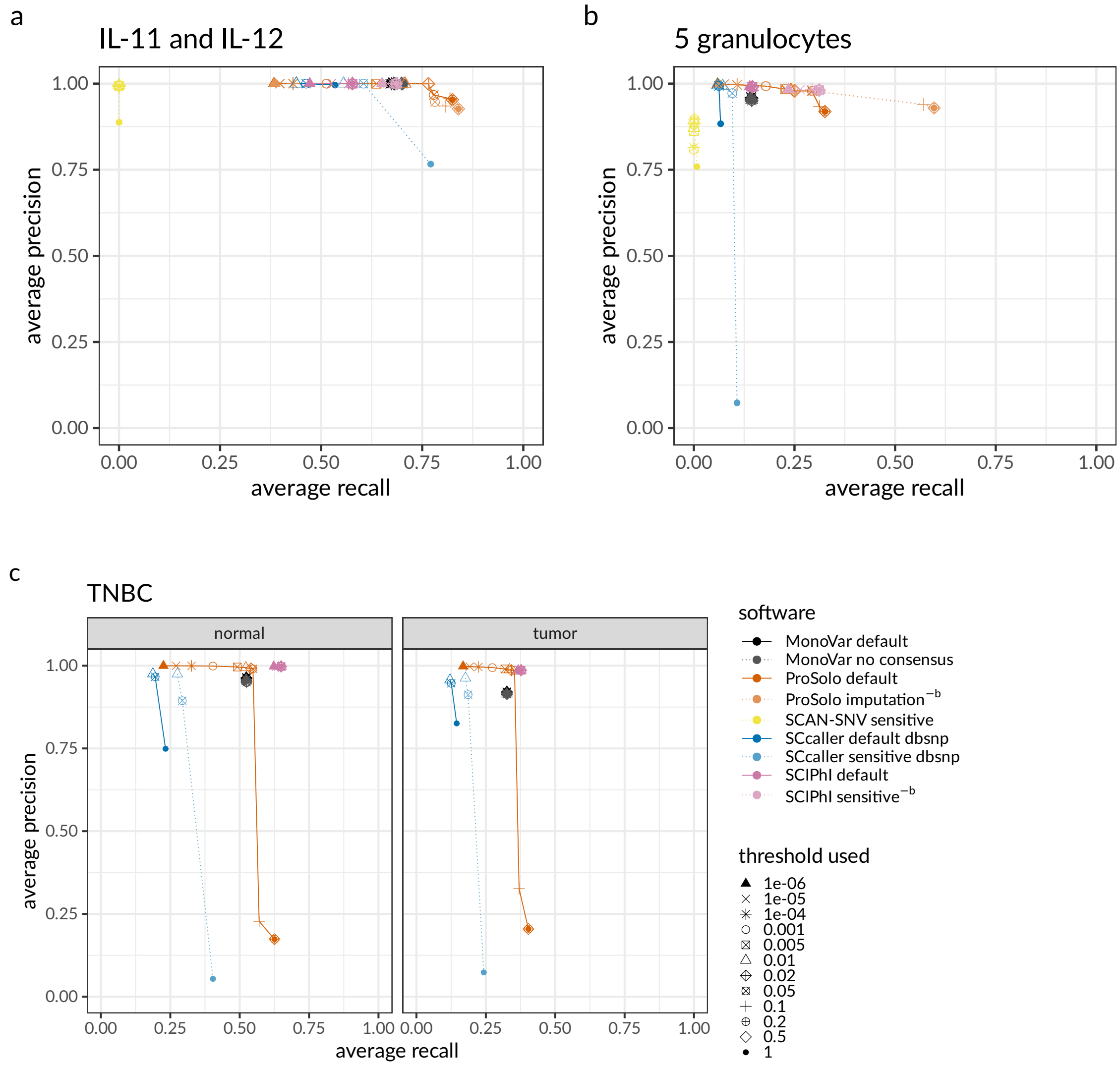

Supplementary Figure 7: Global view of the precision-recall plots for alternative allele calling across different software modes and filtering thresholds (for zoomed in view on the most interesting areas of the plot, see main manuscript Figure 1). **a** Precision and recall average of two whole genome sequenced single cells IL-11 and IL-12 against their kindred clone IL-1c as ground truth genotypes. **b** Precision and recall average of five whole exome sequenced single granulocytes against their pedigree-based germline genotype ground truth.
-b The germline ground truth induces an artificial increase of recall for SCIPhI's sensitive and ProSolo's imputation mode; these modes should thus be disregarded for a fair comparison on the granulocyte dataset in panel b.
c Precision and recall average of 16 tumor and 16 normal single cells sequence at the whole exome level.
Used thresholds are not comparable between tools. They were applied via the following command line options: MonoVar `--t`; ProSolo `--fdr`; SCAN-SNV `--fdr`; SCcaller `-a cutoff`; SCIPhI `prosolo --fdr`. Software modes: MonoVar with consensus filtering (*default*) or without (*no consensus*); ProSolo with minimum coverage 1 in single cell (*default*), or imputing zero coverage sites based on bulk sample (*imputation*); SCcaller with recommended settings (*default*) or with a more *sensitive* calling; SCIPhI with default parameters (*default*) or all heuristics off (*sensitive*).

available for this estimation will be limited and might lead to less reliable estimates. On this dataset, SCAN-SNV's recall increased to .0016% at a decreased maximum precision of .897. Most likely, this decreased precision is an artefact of using the germline genotype as ground truth. At the sites with somatic mutations in single cells, which SCAN-SNV focuses on, this ground truth will instead contain the germline genotype and will incorrectly classify alternative alleles as false positives. Due to this effect, we also expect the calculated precision of all the other tools to be an underestimate, but against the backdrop of large numbers of sites where the single cells harbour the germline genotype, the effect will be much smaller. The same ground truth caveat also applies—inversely—for the recall of SCIPhI's sensitive mode and ProSolo's imputation mode. Whenever coverage of a site is missing in a single cell, SCIPhI may impute the genotype with the last common ancestor genotype of the most closely related cells, while ProSolo will impute to the majority genotype in the bulk sample. While both strategies provide a biologically meaningful imputation that will be more useful than post-hoc modes of imputation, we expect that a number of imputed sites were incorrectly classified as true positives with this germline ground truth. As this will lead to an overestimation of the recall, we have excluded both SCIPhI's sensitive mode and ProSolo's imputation mode from the discussion of this dataset—however, their results are nevertheless displayed in all respective figures.

For the third dataset of single nucleus whole exome sequencing data of 32 cells from a TNBC patient (16 tumor cells, 16 normal cells), we analysed tumor and normal cells separately (Figure 1c). On the tumor cells, ProSolo is the only tool to achieve a precision above .99 (at a recall of .319). On the normal cells, both ProSolo and SCIPhI achieve a precision above .99. Here, SCIPhI outcompetes ProSolo with a maximum recall of .650 compared to ProSolo's .548. While ProSolo can achieve a similar recall (.625, see `--fdr` threshold of 0.2 in Supplementary Figure 7c), this comes at a reduced precision. This reflects the inherent uncertainty of the single cell data and showcases a key difference in

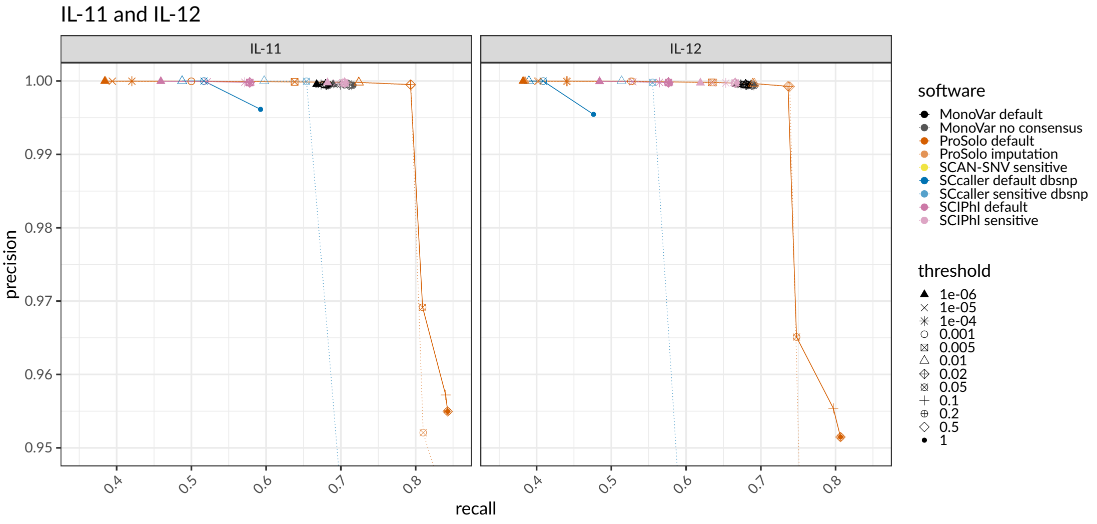

Supplementary Figure 8: Cell-specific precision-recall plots across different filtering threshold levels for the whole genome dataset.

Used thresholds are not comparable between tools. They were applied via the following command line options: MonoVar `--t`; ProSolo `--fdr`; SCAN-SNV `--fdr`; SCcaller `-a cutoff`; SCIPhI `none available`. Software modes: MonoVar with consensus filtering (*default*) or without (*no consensus*); ProSolo with minimum coverage 1 in single cell (*default*), or imputing zero coverage sites based on bulk sample (*imputation*); SCcaller with recommended settings (*default*) or with a more *sensitive* calling; SCIPhI with default parameters (*default*) or all heuristics off (*sensitive*).

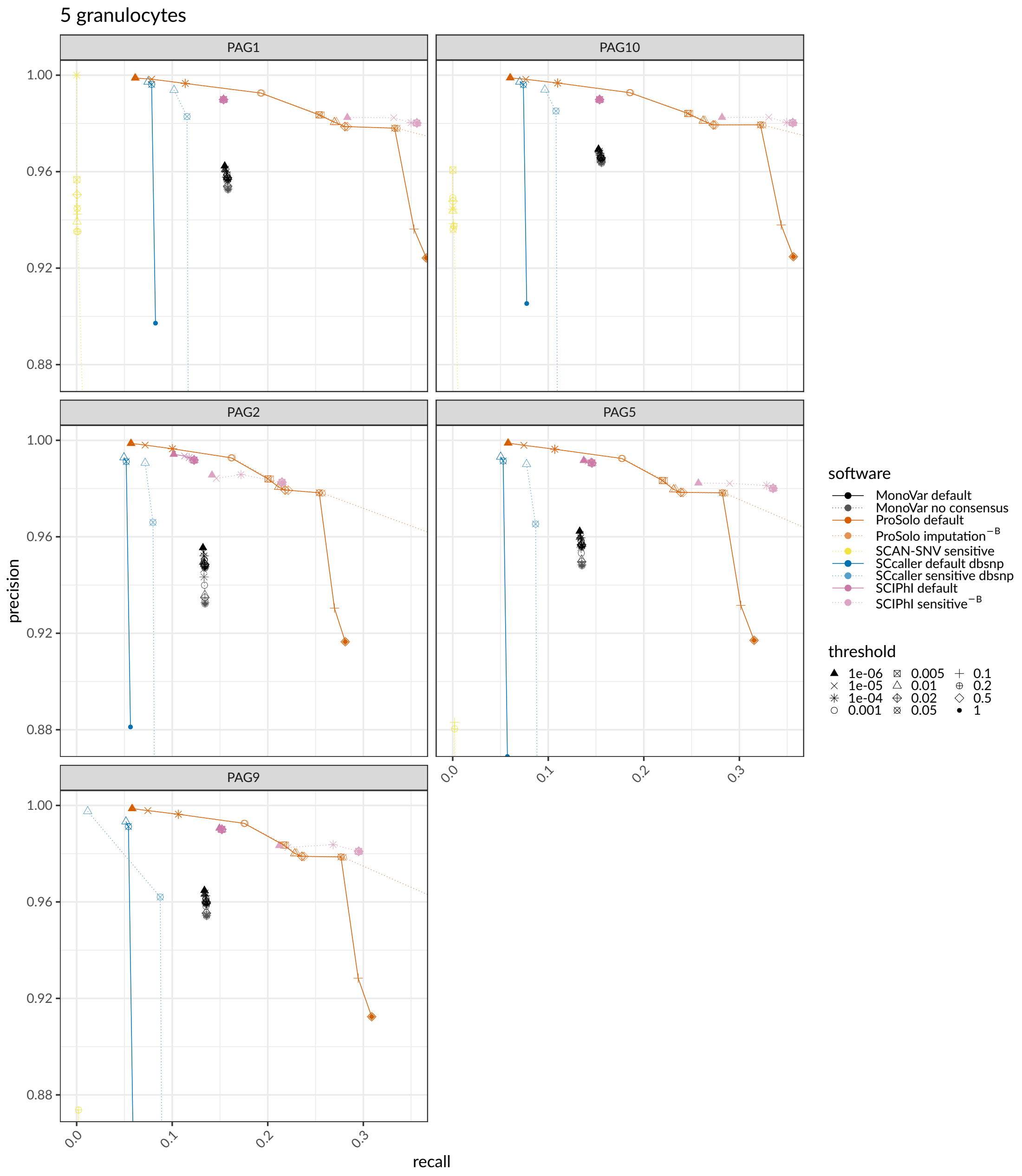

Supplementary Figure 9: Cell-specific precision-recall plots across different filtering threshold levels for the whole exome dataset.

-b The germline ground truth induces an artificial increase of recall for SCIPhI's sensitive and ProSolo's imputation mode; these modes should thus be disregarded for a fair comparison on the granulocyte dataset.

Used thresholds are not comparable between tools. They were applied via the following command line options: MonoVar `--t`; ProSolo `--fdr`; SCAN-SNV `--fdr`; SCcaller `-a cutoff`; SCIPhI `none available`. Software modes: MonoVar with consensus filtering (*default*) or without (*no consensus*); ProSolo with minimum coverage 1 in single cell (*default*), or imputing zero coverage sites based on bulk sample (*imputation*); SCcaller with recommended settings (*default*) or with a more *sensitive* calling; SCIPhI with default parameters (*default*) or all heuristics off (*sensitive*).

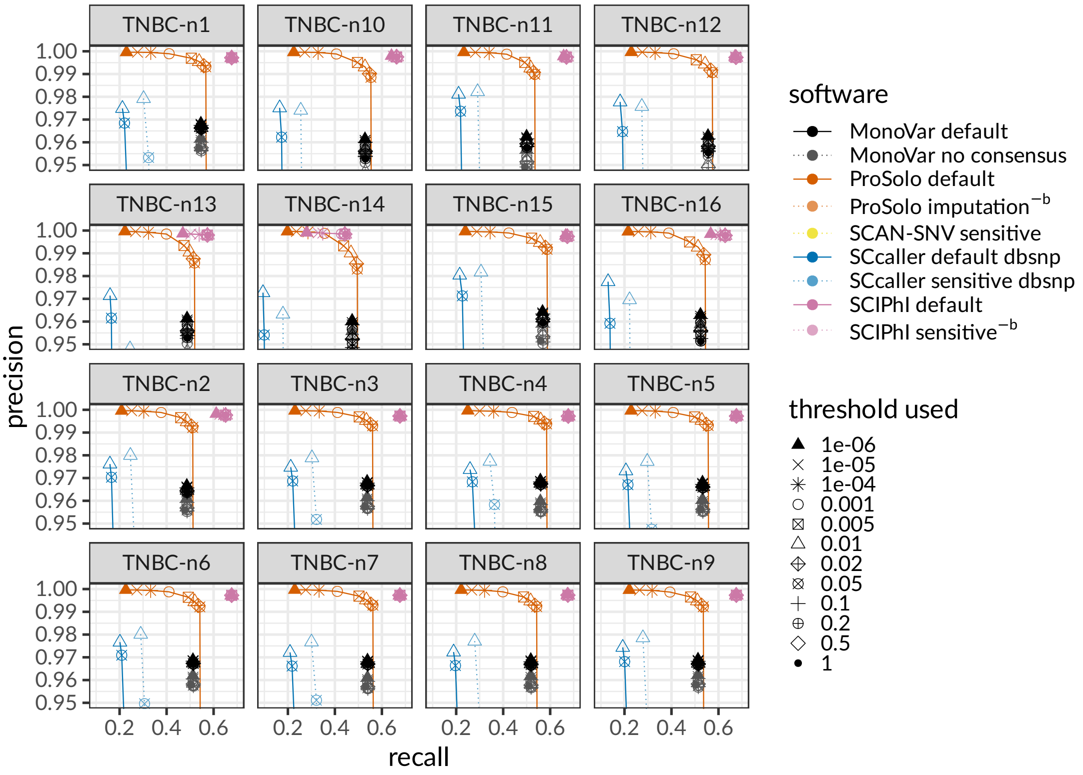

Supplementary Figure 10: Zoomed-in precision and recall per normal cell of the single nucleus exome sequencing dataset Wang et al. [48]. Note cells TNBC-n13 and TNBC-n14 as clear outliers.

For the thresholds used, see caption of Supplementary Figure 7.

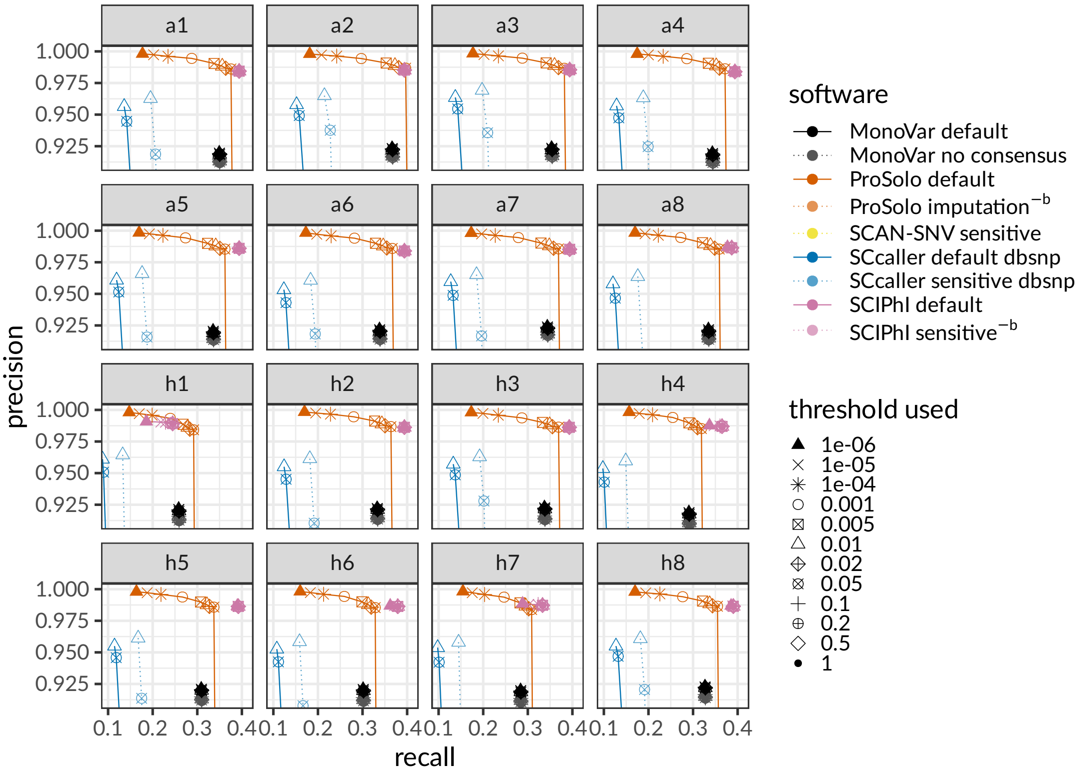

Supplementary Figure 11: Zoomed-in precision and recall per tumor cell of the single nucleus exome sequencing dataset Wang et al. [48]. Note cell h1 as a clear outlier. For the thresholds used, see caption of Supplementary Figure 7.

the approaches of ProSolo and SCIPhI. Where ProSolo models each cell and each genomic site separately, SCIPhI's model integrates information across all sites in all cells at once. While this can clearly help recover recall, this can also lead to false positive somatic variant calls (Supplementary Figure 13) and has clear implications for model complexity and software runtime. As all other tools, including ProSolo, are parallelizable over genomic regions and were thus able to process both datasets within days on a multicore machine, we do not report more detailed runtimes for them. However, it should be noted that SCIPhI took from 1 week (with iterations reduced below software defaults) on the single nucleus whole exome dataset up to 7.5 weeks on the whole genome dataset, running on a single core without any possibility of parallelization. And where adding breadth of coverage (i.e. more genomic sites) or more cells simply means adding more parallel processes in most tools, this will further increase SCIPhI's wall time. In addition, with more cells added, the space of possible tree topologies that SCIPhI explores grows exponentially, further increasing runtimes.

In contrast to the whole genome dataset and the granulocyte whole exome dataset, this dataset showed more variation across cells. To document this, without further conflating the general precision-recall plots, we include per-cell precision-recall plots, here. This shows two outlier cells among the single normal cells, TNBC-n13 and TNBC-n14 (Supplementary Figure 10), and one drastic outlier among the single tumor cells, h1 (Supplementary Figure 10). With h1 at a clearly reduced overall coverage (Supplementary Figure 3C), these outliers—and further more subtle variation across cells—probably reflect variation in the data generation processes rather than biological differences in genomic variation across cells.

2.4 Estimating the bulk contribution to ProSolo results via downsampling

To estimate the importance of bulk data in ProSolo's single nucleotide variant calling model, we performed a downsampling analysis with the whole exome sequencing dataset of five granulocytes. In addition to the full bulk coverage of around 40X at 80% of exome sites (compare Supplementary Figure 3B), we downsampled the bulk sample to around 28X, 16X, 4X, and 0X (Supplementary Figure 12). From this analysis, we can see that even a very minimal coverage of 4X will result in very precise variation calls for any false discovery rate control at 0.1 or below. Only removing the bulk entirely (0X), will significantly impact ProSolo's precision. This is to be expected, as the model does not have any data available to compensate for the uncertainty of amplification bias that the empirical bias distributions account for in the single cell data. In line with this increased uncertainty in variation calls, false discovery rates below 0.2 will remove all calls (Supplementary Figure 12), while the recall remains very high in unfiltered calls.

2.5 Recall of validated somatic variants from Wang et al. [48]

For the tumor cells from the Wang et al. [48] dataset, we could further determine the recall of non-synonymous somatic variants they had validated via deep targeted duplex sequencing and provided in their Supplementary Tables 6 and 7 (specifying clonal and subclonal variants, respectively). Clonal variants were assumed to be present in all 16 tumor cells, with the number of single cells with the subclonal somatic variant present provided by Wang et al. [48]. While SCIPhI and SCcaller have a better recall of heterozygous genotype calls, neither of them can call homozygous alternative genotypes (Supplementary Figure 13). On the clonal variants, MonoVar has a higher homozygous alternative recall than ProSolo, but ProSolo has a higher heterozygous recall. On the subclonal variants, three things are notable:

1. ProSolo has lower recall than the other tools, indicating that the depth of coverage

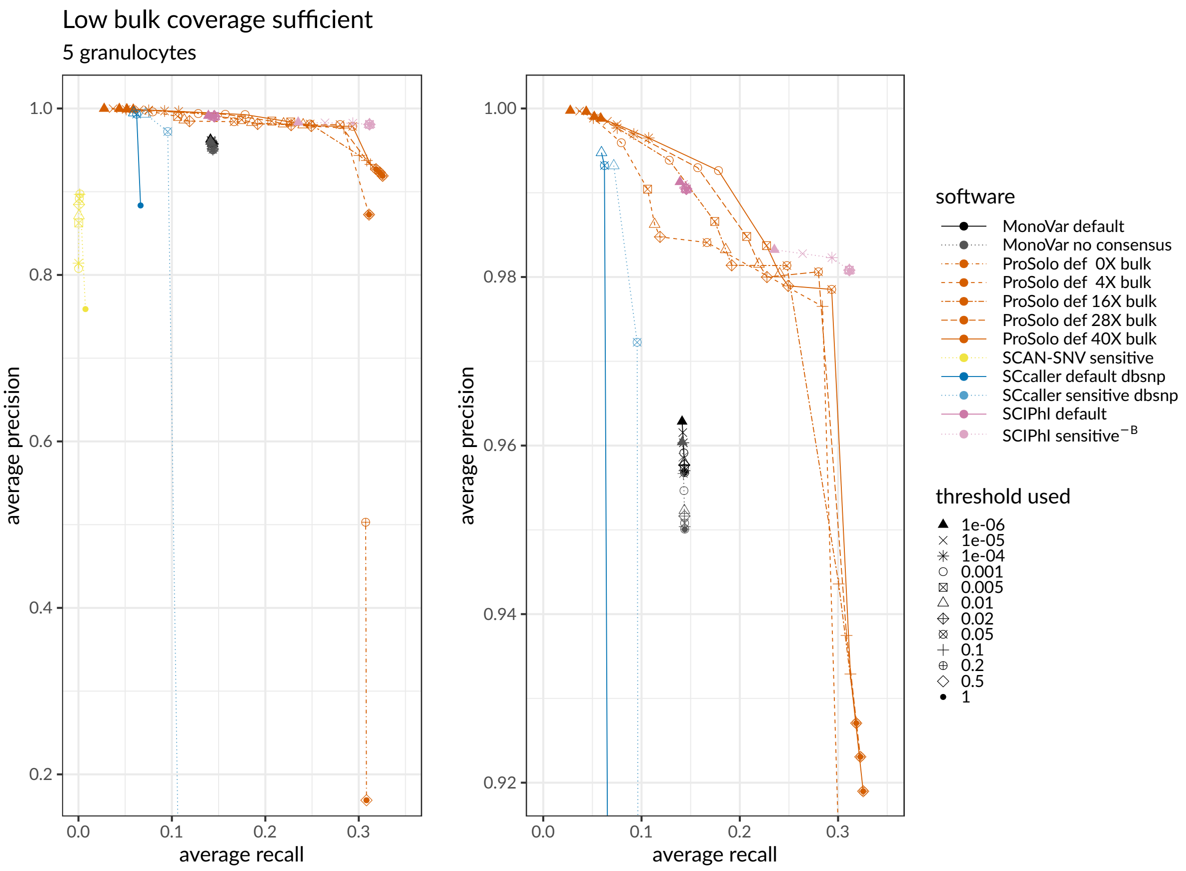

Supplementary Figure 12: Precision and recall average of five whole exome sequenced single granulocytes against their pedigree-based germline genotype ground truth, including the results of ProSolo after downsampling from the maximum coverage of 50X to 35X, 20X, 5X or 0X. The left panel includes all bulk coverages, the right panel focuses on the differences in non-zero bulk coverages.

[-b] The germline ground truth induces an artificial increase of recall for SCIPhI's sensitive and ProSolo's imputation mode; these modes should thus be disregarded for a fair comparison on the granulocyte dataset.

Used thresholds are not comparable between tools. They were applied via the following command line options: MonoVar `--t`; ProSolo `--fdr`; SCAN-SNV `--fdr`; SCcaller `-a cutoff`; SCIPhI `none available`. Software modes: MonoVar with consensus filtering (*default*) or without (*no consensus*); ProSolo with minimum coverage 1 in single cell (*default*), or imputing zero coverage sites based on bulk sample (*imputation*); SCcaller with recommended settings (*default*) or with a more *sensitive* calling; SCIPhI with default parameters (*default*) or all heuristics off (*sensitive*).

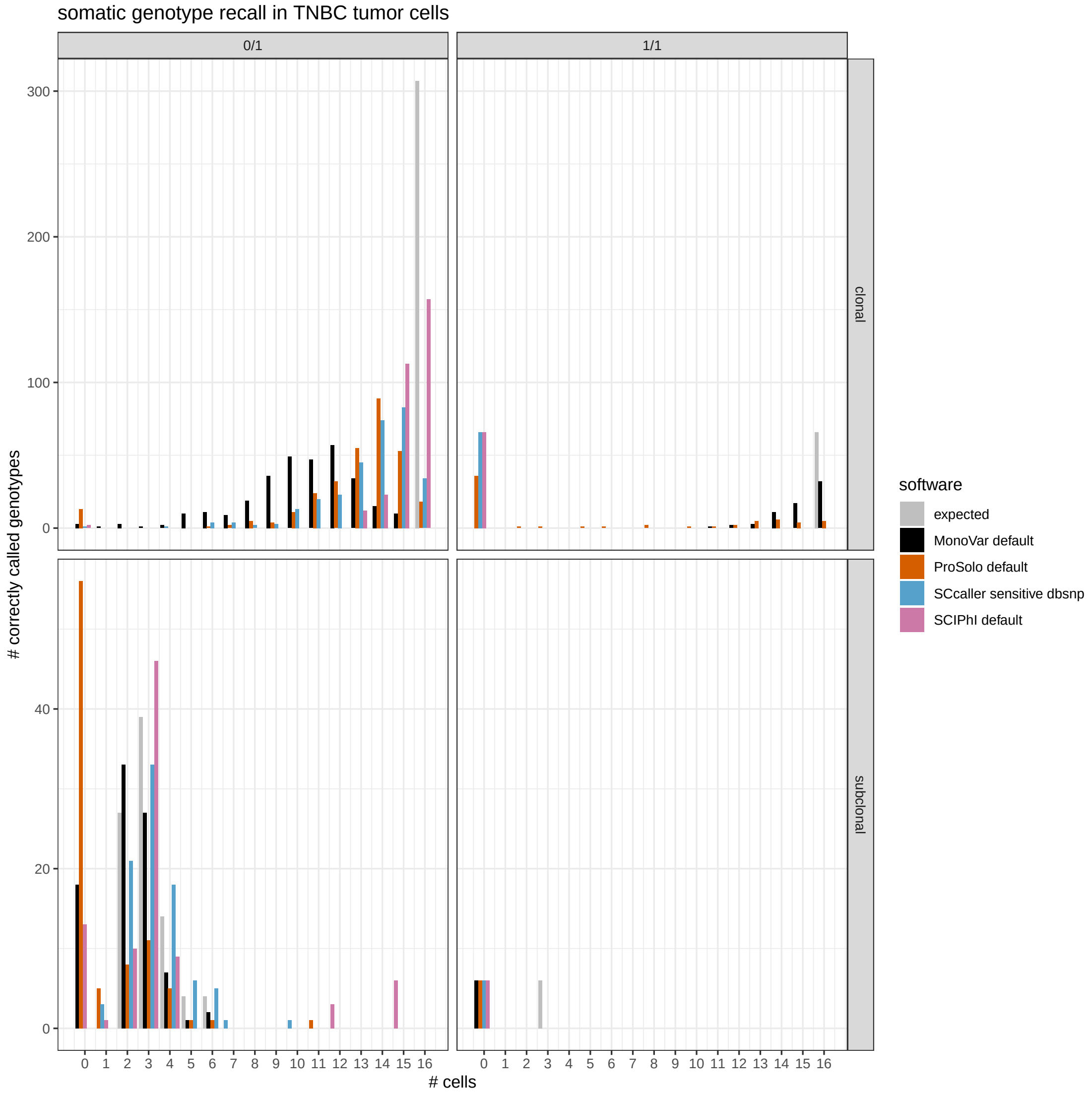

Supplementary Figure 13: Recall of validated non-synonymous somatic mutations in the single tumor cells of Wang et al. [48] stratified across genotypes ("0/1" for heterozygous and "1/1" for homozygous alternative) and clonality (clonal vs. subclonal). The expected number of variants with a somatic variant present in the respective number of single tumor cells is given in grey. Clonal somatic variants are always expected to be present in all 16 single tumor cells.

Supplementary Table 4: Genomic sites where any tool calls the presence of a variant in at least three more cells than the number reported in Wang et al. [48]. "Wang duplex freq" gives the alternative allele frequency from the targeted duplex bulk sequencing (deep coverage and high accuracy) in Wang et al. [48]. All variants in this table are reported as heterozygous by Wang et al. [48]. Results are from each software's best performing software mode without any threshold-based filtering.

* MonoVar called 5 cells as heterozygous and 4 cells as homozygous alternative

			number of cells with variant (out of 16)				
chromosome	position	Wang duplex freq	Wang	MonoVar	ProSolo	SCcaller	SCIPhI
chr16	1537917	0.102820	3	3	0	4	12
chr16	15149761	0.057895	2	3	0	3	12
chr16	66643819	0.189566	5	6	6	6	15
chr17	77809062	0.001294	2	3	0	3	15
chr2	74435818	0.091089	3	4	0	3	12
chr5	55204134	0.233289	6	*9	11	10	15
chr9	19298104	0.131816	4	6	0	6	15
chr9	80046280	0.000269	2	2	0	2	15
chr9	117853210	0.001309	4	3	3	4	15

in the tumor bulk sample (around 35X for 80% of the exome, see Supplementary Figure 3C) is not sufficient to pick up low-frequency variants and the bulk sample thus further decreases the likelihood of such low frequency variants in the single cells. Thus, while the downsampling analysis above suggests that a very low bulk coverage is sufficient for ProSolo's model to perform well, this seems to be restricted to higher frequency mutations. Assuming an evenly mixed cell population (thus ignoring any population structure or in turn assuming an even sampling of all relevant subclones), the bulk coverage of 35X should allow for the discovery of variant alleles present at a frequency of at least 2.86% of sampled alleles. Judging by the frequency detected in the targeted duplex sequencing of Wang et al. [48], this should render the detection of 52 of the 94 subclonal variants impossible, which corresponds to the number of missed subclonal variants in Supplementary Figure 13. As a consequence, if the aim is to detect lower frequency mutations, we simply recommend a deeper sequencing of the bulk sample.

2. SCIPhI calls nine heterozygous mutations in 12 or 15 cells (Supplementary Table 4 and Supplementary Figure 13), while the maximum frequency for any of the heterozygous subclonal mutations detected in the deeply sequenced tumor bulk was 0.233, more in the range of the maximum number of six out of 16 cells that Wang et al. [48] found any of the subclonal variants in. Also, when able to detect the variant, all other tools report a maximum excess of two cells with the mutation compared to Wang et al. [48]. The only exception is a mutation on chromosome five, where all tools agree on a much larger number of cells with the mutation (9-11) than originally reported by Wang et al. (6), but SCIPhI still clearly exceeds that consensus (15). Altogether, these numbers suggest that SCIPhI's model overimputes mutations by aggregating information across cells.

3. None of the tools calls any of the six homozygous alternative variants. SCcaller

and SCIPhI do not genotype and thus can only determine the presence or absence of an alternative allele. We chose to represent this as a heterozygous alternative genotype, as is this will be the correct choice in the majority of cases. For MonoVar, this is surprising, as all these homozygous alternative variants should be present in three cells, which should allow MonoVar to recover them even when enforcing an occurrence in a minimum of three independent single cells. For ProSolo, this seems to be due to the low bulk coverage of these variants. The low coverage of these variants in the duplex sequencing means that the alternative allele is probably not covered in the low coverage bulk tumor sample at all, and ProSolo accordingly assigns a homozygous reference genotype. However, if the bulk coverage were higher, ProSolo might still suffer from its assumption of a maximum of two different genotypes at a site in the bulk sample. This can lead to erroneously mis-calling homozygous alternative sites as heterozygous, when they are the result of a point mutation followed by a loss of the reference allele in a daughter cell (Supplementary Section 1.5.1). However, an even simpler explanation (for all tools) could be that the ground truth genotype was called with GATK UnifiedGenotyper, a variant caller that is not adapted to single cell DNA-sequencing data, and may have thus suffered from allele dropout of the reference allele to generate these homozygous alternative genotypes. This could mean that these homozygous alternative ground truth are not correct to begin with.

2.6 False Discovery Rate Control of Alternative Allele Calling

Finally, a feature where ProSolo clearly stands out, is the control over the false discovery rate. As can be seen in Figure 1 (and Supplementary Figure 7), ProSolo provides flexible control over precision vs. recall via specifying a false discovery rate of interest. While no other tool provides a formal control over the easily interpretable false discovery rate, several of the tools provide other types of thresholds which we varied in attempts to

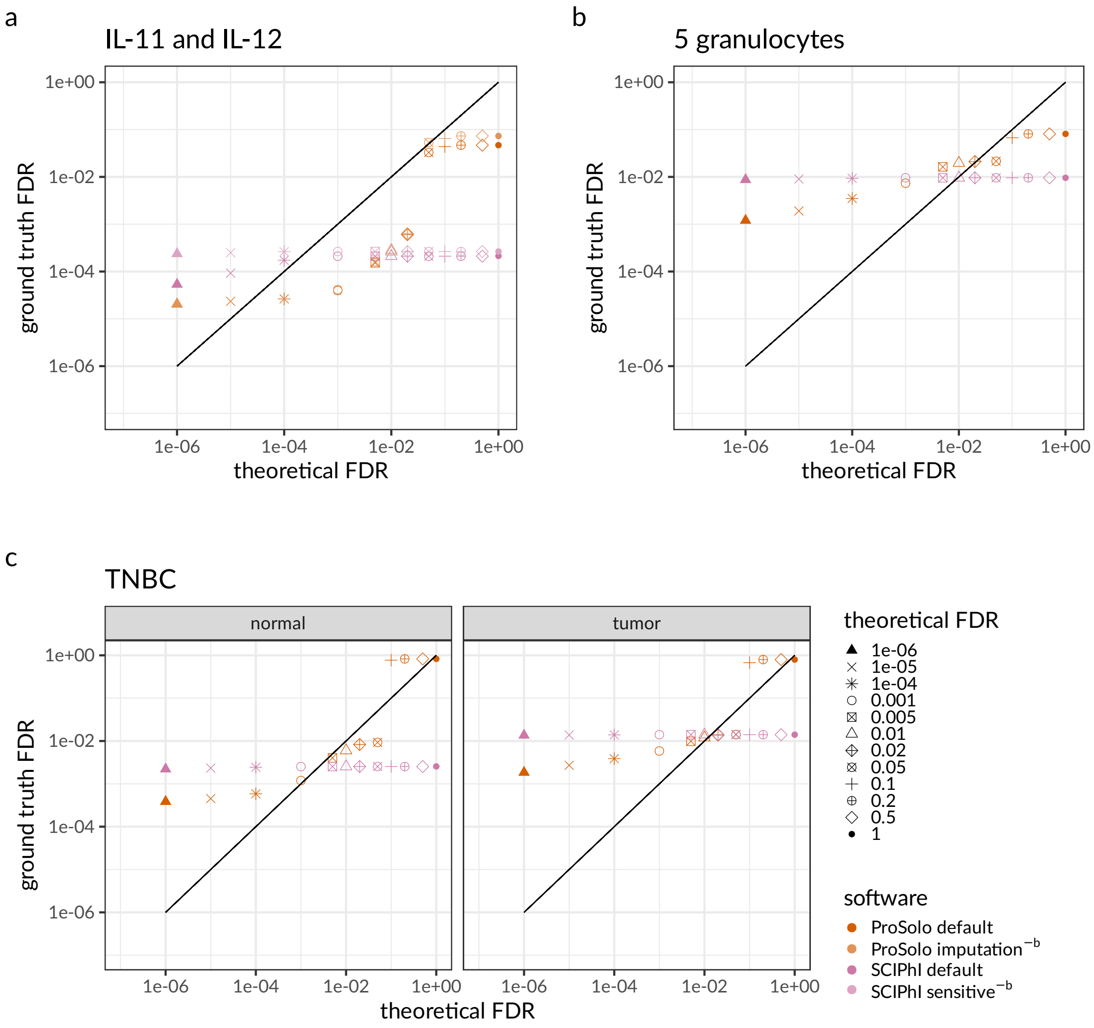

Supplementary Figure 14: False discovery rate (FDR, $1-$precision) as calculated based on the ground truth, compared to the theoretical FDR we controlled for using ProSolo, for **a** the whole genome cell-line data set, **b** the whole exome data set with the pedigree ground truth and **c** the whole exome cancer data set. The diagonal indicates identity of the ground truth FDR with the FDR that was controlled for. Any points underneath it indicate overly conservative FDR control, any points above it indicate an FDR control that is too lenient.

Used thresholds are not comparable between tools. They were applied via the following command line options: MonoVar `--t`; ProSolo `--fdr`; SCAN-SNV `--fdr`; SCcaller `-a cutoff`; SCIPhI `none available`. Software modes: MonoVar with consensus filtering (*default*) or without (*no consensus*); ProSolo with minimum coverage 1 in single cell (*default*), or imputing zero coverage sites based on bulk sample (*imputation*); SCcaller with recommended settings (*default*) or with a more *sensitive* calling; SCIPhI with default parameters (*default*) or all heuristics off (*sensitive*).

achieve higher precision or recall. However, none of them provide control over similar ranges of precision and recall. In addition, we ran the standard false discovery rate control implemented in ProSolo on the posterior probabilities provided by SCIPhI, but this did not provide any substantial control over the false discovery rate (Figures 1 and 14). Thus, ProSolo is the only tool that provides the user with the choice of either aiming for more discoveries at the cost of a higher rate of false discoveries, or at aiming for a more limited number of discoveries with higher confidence in each of them.

Further, Supplementary Figure 14a demonstrates, that ProSolo provides a rigorous control over the false discovery rate on the whole genome dataset. The only exception—in both default and imputation mode—is when attempting to control for a false discovery rate below 0.0001. This indicates, that ProSolo's model still needs refinement in its resolution for sites with a very high likelihood of an alternative allele present. The same trend can be seen on the other datasets (Supplementary Figure 14b and c), but for any attempt to control for a false discovery rate below 0.02 or 0.005. This is within the common range of values often chosen as a false discovery rate to control for in practice and not being able to actually achieve the false discovery rate control at such values is usually unacceptable. However, as also discussed above, we generally expect the precision (of all tools) to be underestimated on the whole exome granulocyte dataset: using the germline genotypes as ground truth results in misclassifying actual somatic variants in single cells as false positives. This underestimation of precision on this dataset in turn means an overestimation of the false discovery rate ($1 - p$recision), and is thus to be expected. A similar effect is to be expected for the tumor cells in the TNBC dataset, as these will very likely contain more than the clonal somatic variants validated in the original publication [48]. Further, the artefact of using a germline ground truth on the granulocyte dataset also shows in the discrepancy in the ground-truth based false discovery rate between ProSolo's default and imputation mode when controlling for higher false discovery rates. As expected, imputation turned out less precise in the

whole genome dataset (Supplementary Figure 14a). In contrast, imputation seems to have improved precision for the whole exome dataset (Supplementary Figure 14b). This would suggest that the larger call set from ProSolo's imputation mode (with a higher recall) is also more precise than the smaller call set from the default mode. However, this increase in precision is most likely explained by an imputation towards the majority genotype in the bulk sample that will usually favor the germline genotype at a site, no matter what the actual single cell genotype would be. Taken together, this indicates that the false discovery rate that we can calculate with the given germline ground truth for the whole exome dataset is higher than the actual value would be with a more accurate (somatic) ground truth available.

2.7 Allele dropout rate calculations

Leveraging our ground truths and using three different ways to calculate the allele dropout rate, we can confirm the general validity of the respective event definitions (Figure 3D) and evaluate limitations. For this, we will focus on the set of sites where the respective ground truth call is heterozygous, as these are the sites where the dropout of one of the alleles can be identified clearly.

The first way of calculating the allele dropout rate leverages the posterior probabilities generated by the ProSolo model (Equation S 31). At each ground truth heterozygous site, we sum up the probabilities of the two allele dropout events defined in ProSolo ("ADO to alt" and "ADO to ref" in Figure 3D) to obtain a total allele dropout probability (see Equation S 31). Obtaining the expected value of the allele dropout rate then constitutes adding those totals across all ground truth heterozygous sites with coverage and dividing by their number. This gives us an expected allele dropout rate based on the data and the ProSolo model ("expected value" in Figure 2):

$$\frac{\displaystyle\sum_{\substack{\text{covered ground truth}\\ \text{heterozygous sites}}} P(\text{allele dropout} \mid \boldsymbol{Z}^s, \boldsymbol{Z}^b)}{\#\ \text{covered ground truth heterozygous sites}} \tag{S 33}$$

The two ways to validate this expected allele dropout rate both depend on a comparison against the respective ground truth genotypes. One important difference in the ground truth used here, compared to the ground truth used for alternative allele calling, is that we further filtered the ground truth for the Dong et al. [11] dataset. For this dataset, the basic ground truth was generated using GATK HaplotypeCaller and variant quality score recalibration. However, this recalibration optimises on the presence (heterozgyous or homozygous alternative) vs. the absence (homozygous reference) of an alternative allele and filtered genotypes will not reliably distinguish between heterozygous genotypes and homozygous alternative genotypes. We thus excluded all sites where the genotype quality (`GQ` FORMAT field) is below a PHRED score of 30. This corresponds to a 0.001 probability of having erroneously called the current genotype over the second most likely genotype, and thus also filters for a clear distinguishability between heterozygous and homozygous alternative genotypes. This is important both for identifying allele dropout and for identifying exact genotypes as in Section 2.8.

The first validation of these rates from within ProSolo is to genotype all the ground truth heterozygous sites with ProSolo. We take the most likely genotype (i.e. the genotype whose coloured event area in Figure 3D has the highest joint probability). Then, every ground truth heterozygous site that ProSolo calls as homozygous is counted as a dropout site and the number of dropout sites is divided by the total number of ground truth heterozygous sites where the respective single cell had coverage. This gives an allele dropout rate based on a comparison against the ground truth ("hom at ground truth het" in Figure 2):

$$\frac{\#\text{ ground truth heterozygous sites called homozygous}}{\#\text{ covered ground truth heterozygous sites}} \tag{S 34}$$

A second validation of these rates is another comparison against the ground truth, but without using ProSolo in any way. For each single cell, using samtools mpileup, we identify all heterozygous ground truth sites with a coverage of at least 7. Without amplification bias we could be reasonably sure to sample both alleles of a heterozygous site at coverage 7 (the probability to sample only one allele with a coverage of 7 would be: $2 \cdot 0.5^7 = 0.015625$). We then count a site as an allele dropout, if there is one allele (reference or alternative) with no read coverage at all. The allele dropout rate is the fraction of allele dropout sites within the respective cell's heterozygous ground truth sites (”no alt/ref read at ground truth het” in Figure 2):

$$\frac{\#\text{ ground truth heterozygous site with only one allele covered}}{\#\text{ covered ground truth heterozygous sites}} \tag{S 35}$$

The expected allele dropout rates based on the ProSolo probabilities for allele dropout fall into the range of previously published allele dropout rates [48, 15, 51, 27, 45, 40, 28] (”published” in Figure 2). This analysis also shows that the ProSolo expected allele dropout rates, based on the model's probabilities, correspond with those based on the comparison of ProSolo genotypes with the ground truth (Figure 2), demonstrating that the explicit modelling of allele dropout events is useful for genotyping. However, in that comparison, the expected allele dropout rate is consistently underestimated on our own whole exome data (”granulocytes” in Figure 2), while it is slightly overestimated for the data from Dong et al. [11] (”Dong 2017” in Figure 2). These deviations in the

estimation in different directions suggest that the empirical distributions we currently use do not fit all datasets. Instead, they most likely depend on the respective whole genome amplification and library preparation procedures, as well as other possible batch effects. This conclusion is further supported by the much lower naively calculated allele dropout rate in the high coverage cells of the two datasets (IL-11 in Dong et al. [11], PAG1 and PAG10 in our granulocytes, compare 3). Here, the empirical distributions used seem to overestimate allele dropout and the performance of ProSolo could be further improved with distributions tailored to the specific dropout profile of the experiment on that individual cell. However, this overestimation of the allele dropout probability does not seem to impact the genotyping resolution (see below, Figures 15 and 16).

2.8 Genotype calling

As a further performance comparison, we want to include a direct comparison of the actual called genotypes against the ground truth genotypes of the respective data set. As only ProSolo, MonoVar and SCAN-SNV provide actual genotype calls—a further refinement over the alternative allele calling described above—SCcaller and SCIPhI cannot be included in this comparison. Further, the small numbers of calls made by SCAN-SNV would not be interpretable in the figures provided, and we have thus also excluded SCAN-SNV from this comparison.

Comparisons provided here are based on the software modes that achieved the highest F_1 score across most single cells for alternative allele calling (Figure 1c), with the F_1 score defined as the harmonic mean of precision p and recall r (with $\beta = 1$ for the general F score, see van Rijsbergen [47], Chinchor [7]):

$$F_\beta = (\beta^2 + 1) \times \frac{pr}{\beta^2 p + r} \tag{S 36}$$

.

For ProSolo, this amounts to the default mode without imputation (which also ensures a fair comparison against the germline-based ground truth) and controlling for a false discovery rate of 0.2. For MonoVar, this means no consensus filtering and no filtering via the `--t` thresholding parameter.

Plotting the overall precision and recall of ProSolo and MonoVar genotyping (Supplementary Figure 15), demonstrates that the higher recall of ProSolo is limited to the calling of heterozygous genotypes for the whole genome datasets (IL-11, IL-12 in Supplementary Figure 15A). For the whole exome dataset, ProSolo achieves a higher recall of both heterozygous and homozygous alternative ground truth genotypes (PAG cells in Supplementary Figure 15A). However, the recall remains much lower in this dataset, possibly due to the more uneven coverage of the respective cells and the lower coverage of the background bulk sample (PNG in Supplementary Figure 3).

Wherever ProSolo generates more genotype calls—be they heterozygous or homozygous alternative calls—it also makes more errors (Supplementary Figure 15B). In the only case where ProSolo does not generate more calls than MonoVar—homozygous alternative calls in the whole genome datasets—it also does not show a noticeable increase in false genotype calls.

When looking at the exact genotype miscalls (Supplementary Figure 16), it becomes apparent that the higher amount of miscalls at higher recall in ProSolo is biased for heterozygous calls made by ProSolo (Supplementary Figure 16B): Heterozygous calls made by ProSolo are more often at ground truth homozygous reference sites, whereas heterozygous calls at ground truth homozygous alternative sites do not occur more often than for MonoVar. Thus, while MonoVar heterozygous calls are more likely to be miscalled homozygous alternative sites than miscalled homozygous reference sites, the inverse holds for ProSolo. This indicates, that both models are not yet well-balanced. For ProSolo, a further improvement could be to adapt the amplification bias distribu-

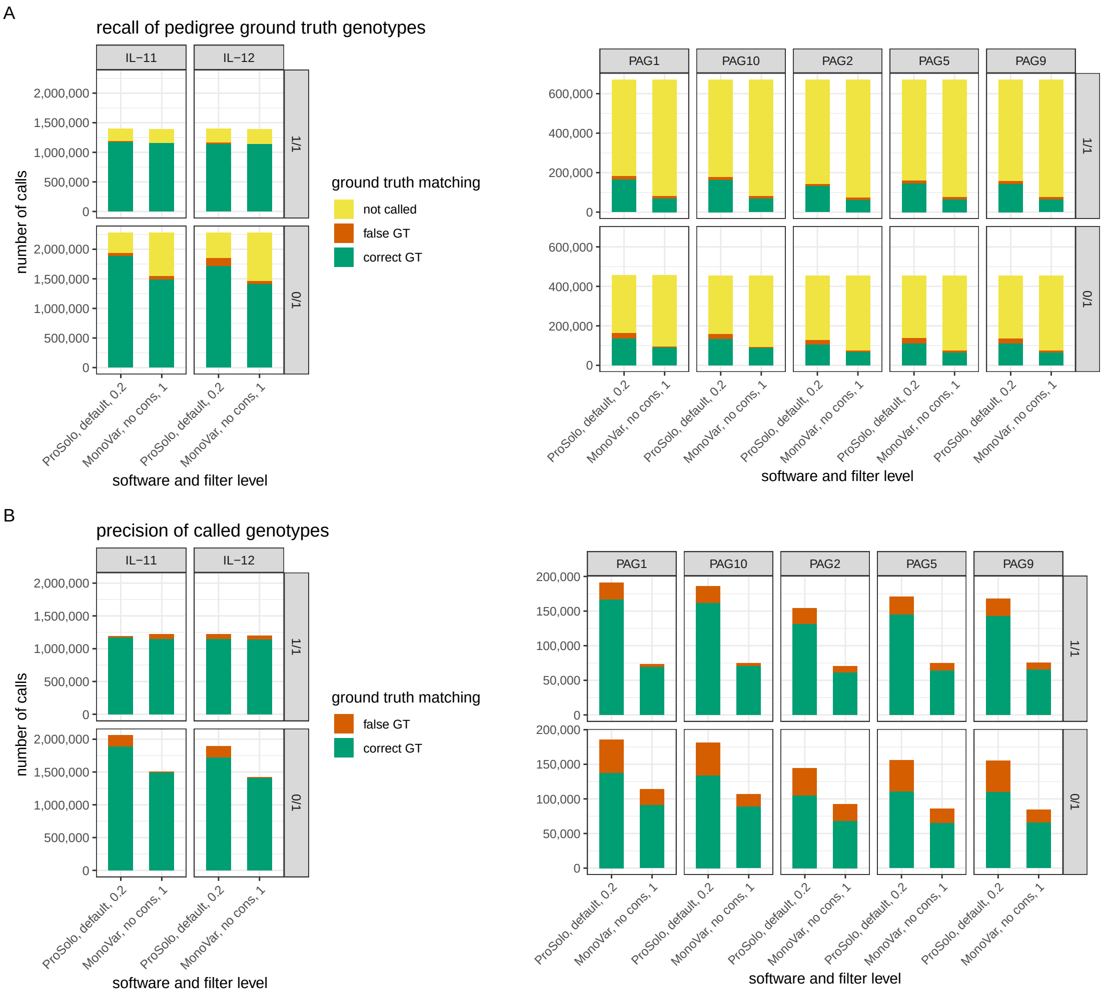

Supplementary Figure 15: Genotype calling precision and recall in comparison against the respective ground truth genotypes (from IL-1c in the left plots; from PNG in the plots on the right). MonoVar and ProSolo were run with the parameters that maximised the F_1 score alternative allele calling (Figure 1c). Facet labels at the top of plots indicate the single cell. Facet labels on the right side of plots indicate the genotype: "1/1" for homozgyous alternative, "0/1" for heterozygous. **A** Recall of ground truth genotypes. Here, facet labels on the right side of plots indicate the ground truth genotype compared against. ProSolo provides a genotype call at more of the ground truth sites, with a slight but noticeable increase of false genotypes provided at ground truth heterozygous sites. **B** Precision of called genotypes. Here, facet labels on the right side of plots indicate the genotype of the caller. The higher amount of calls by ProSolo (most noticeable for heterozygous calls for the whole genome datasets, and homozygous alternative calls for the whole exome datasets) trades off with a loss in precision.

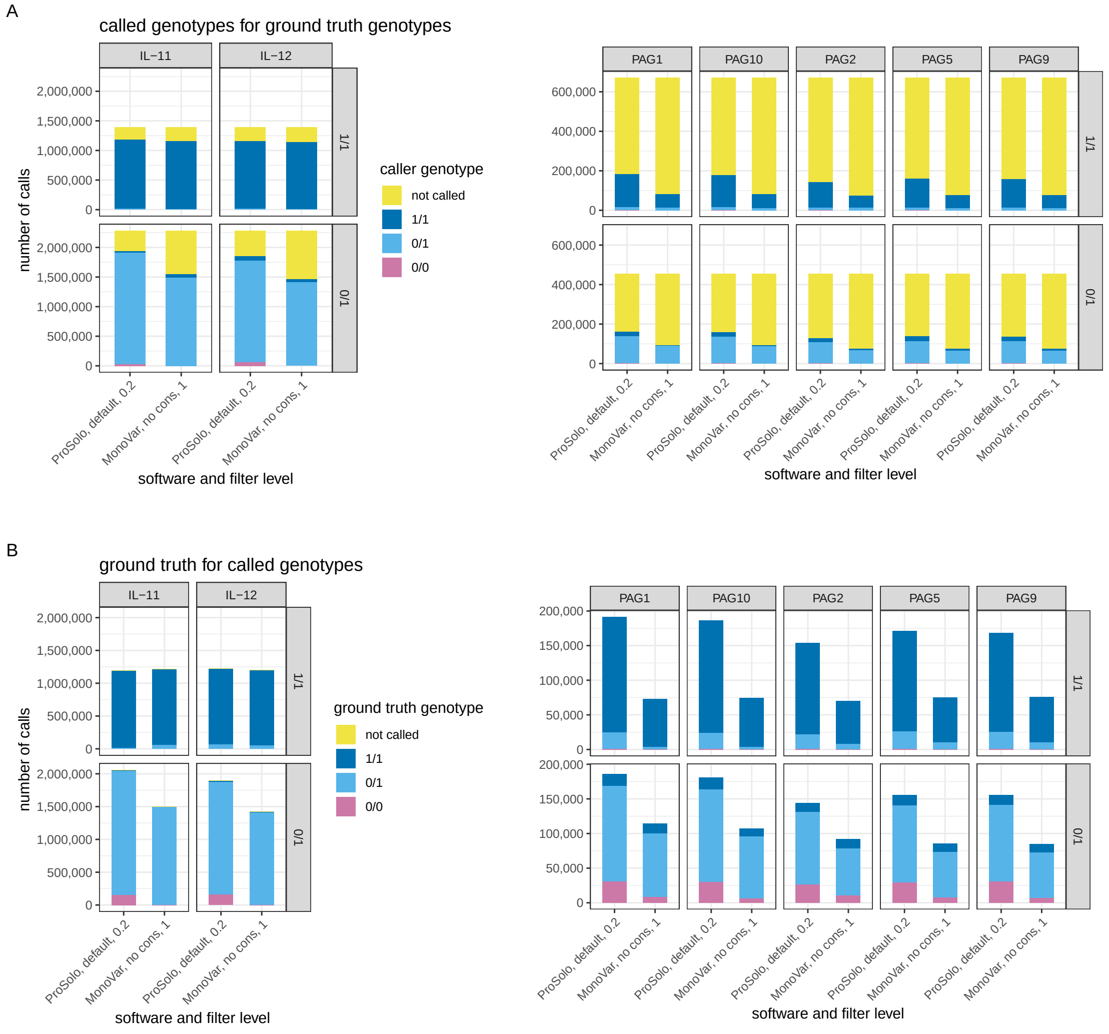

Supplementary Figure 16: Genotype calling comparison against the respective ground truth genotypes (from IL-1c in the left plots; from PNG in the plots on the right). MonoVar and ProSolo were run with the parameters that maximised the F_1 score alternative allele calling (Figure 1c). Panels A and B correspond to the respective panels in Supplementary Figure 15, but with a finer resolution of the false genotypes. Facets at the top of plots indicate the single cell. **A** Genotypes called by the respective tool are indicated by the colouring, for the respective ground truth genotypes as indicated by the facets on the right side of the plots. **B** Ground truth genotypes are indicated by the colouring, for the genotypes called by the respective tool as indicated by the facets on the right side of the plots. Both tools show visible amounts of misclassification in most of the possible categories.

tions currently used (see Section 1.2.2 for some suggestions). Also, learning distributions from the data at hand could further improve precision and recall wherever data is more uniformly distributed than in the dataset the current distributions are derived from [28].

Altogether, the genotype-level analysis also confirms that a higher and more uniform coverage of the single cell data leads to higher precision and recall in the variant calling, no matter which caller is used. E.g. for the—most evenly covered—whole genome sequencing data (Supplementary Figure 3A), both callers achieve a very high recall of ground truth homozygous alternative sites with a very high precision (left panels in Figures 15 and 16). Also, for the whole exome data (right panels in Figures 15 and 16), recall is visibly higher for all genotypes across both tools in the samples with a higher and more uniform coverage (PAG1 and PAG10, see Supplementary Figure 3), and precision is noticeably higher in those samples for MonoVar.

2.9 Software and Resources used

All analysis code for both the main processing pipeline and the manuscript figures is available at: `https://github.com/prosolo/benchmarking_prosolo` (or as preserved by Zenodo at: `https://doi.org/10.5281/zenodo.3769115`). An overview and citations of software and resources beyond those mentioned in the methods section are provided in Supplementary Table 5.

Supplementary Table 5: Software and related resources used in this manuscript. All software versions as installed with conda, except: Kaluza and Summit, which were pre-installed on the fluorescence activated cell sorting machine; Brewer and Okabe Ito, which are color palettes; bioconda, which is a package repository for the conda (and mamba) package manager.

resource	version	purpose	citation
bcftools	1.6<br>1.10	handle VCF / BCF files<br>rule-specific environment	Danecek et al. [9]
BEAGLE 4.0	4.0	pedigree-aware variant calling	Browning and Browning [6]
bioconda		software package management	Grüning et al. [13]
Brewer		colorblind-friendly palettes	Brewer [5]
conda	4.9.0	software package management	
cutadapt	1.14	adapter trimming	Martin [30]
bwa mem	0.7.15	read alignment / mapping	Li [25]
FamSeq	1.0.3	pedigree-aware variant calling	Peng et al. [37, 38]
ggplot2	3.3.0	result plots	Wickham [49]
ggthemes	0.2.5	Okabe Ito palette	Arnold et al. [1]
Inkscape	0.92.4	info-graphics, improved labels	
Kaluza	2.1	FACS data analysis	
mamba	0.6.4	software package management	
MonoVar	0.0.1	single cell variant calling	Zafar et al. [55]
mosdepth	0.2.5	coverage calculations	Pedersen and Quinlan [36]
Okabe Ito		colorblind-safe palette	Okabe and Ito [35]
picardtools	2.9.2	mark duplicates	
polymutt	0.18	pedigree-aware variant calling	Li et al. [24]
ProSolo	0.6.1	single cell variant calling	
RColorBrewer	1.1_2	Brewer palettes	Neuwirth [34]
samtools	1.4.1	handle SAM / BAM files	Li et al. [26]
SCAN-SNV	19-10-30	single cell variant calling	Luquette et al. [29]
SCcaller	1.2	single cell variant calling	Dong et al. [11]
SCIPhI	0.1.4	single cell variant calling	Singer et al. [44]
Snakemake	5.4.0	workflow management	Köster and Rahmann [20]
Summit	5.1.0	FACS data acquisition	
tidyverse	1.1.1<br>1.3.0	ground truth comparisons<br>results data handling	Wickham et al. [50]
trimmomatic	0.36	quality trimming	Bolger et al. [4]
UpsetR		set intersection plot 5	Conway et al. [8]
VGAM	1.1_1	distributions in R	Yee [52, 53]

Supplementary References

[1] J. B. Arnold, G. Daroczi, B. Werth, B. Weitzner, J. Kunst, B. Auguie, B. Rudis, H. W. C. f. t. g. package.), J. T. C. f. t. l. package), and J. London. ggthemes: Extra Themes, Scales and Geoms for 'ggplot2', May 2019. URL https://CRAN.R-project.org/package=ggthemes.

[2] T. Baslan, J. Kendall, L. Rodgers, H. Cox, M. Riggs, A. Stepansky, J. Troge, K. Ravi, D. Esposito, B. Lakshmi, M. Wigler, N. Navin, and J. Hicks. Genome-wide copy number analysis of single cells. *Nature Protocols*, 7(6):1024–1041, June 2012. ISSN 1754-2189, 1750-2799. doi: 10.1038/nprot.2012.039. URL http://www.nature.com/articles/nprot.2012.039.

[3] Y. Benjamini and Y. Hochberg. Controlling the False Discovery Rate: A Practical and Powerful Approach to Multiple Testing. *Journal of the Royal Statistical Society. Series B (Methodological)*, 57(1):289–300, 1995. URL http://www.jstor.org/stable/2346101.

[4] A. M. Bolger, M. Lohse, and B. Usadel. Trimmomatic: a flexible trimmer for Illumina sequence data. *Bioinformatics*, 30(15):2114–2120, Aug. 2014. ISSN 1367-4803. doi: 10.1093/bioinformatics/btu170. URL https://academic.oup.com/bioinformatics/article/30/15/2114/2390096. Publisher: Oxford Academic.

[5] C. Brewer. ColorBrewer: Color Advice for Maps, 2006. URL http://colorbrewer2.org/#.

[6] B. L. Browning and S. R. Browning. Improving the Accuracy and Efficiency of Identity-by-Descent Detection in Population Data. *Genetics*, 194(2):459–471, June 2013. ISSN 0016-6731, 1943-2631. doi: 10.1534/genetics.113.150029. URL http://www.genetics.org/content/194/2/459.

[7] N. Chinchor. MUC-4 Evaluation Metrics. In *Proceedings of the 4th Conference on Message Understanding*, MUC4 '92, pages 22–29, Stroudsburg, PA, USA, 1992. Association for Computational Linguistics. ISBN 978-1-55860-273-1. doi: 10.3115/1072064.1072067. URL https://doi.org/10.3115/1072064.1072067. event-place: McLean, Virginia.

[8] J. R. Conway, A. Lex, and N. Gehlenborg. UpSetR: an R package for the visualization of intersecting sets and their properties. *Bioinformatics*, 33(18):2938–2940, Sept. 2017. ISSN 1367-4803. doi: 10.1093/bioinformatics/btx364. URL https://academic.oup.com/bioinformatics/article/33/18/2938/3884387.

[9] P. Danecek, A. Auton, G. Abecasis, C. A. Albers, E. Banks, M. A. DePristo, R. E. Handsaker, G. Lunter, G. T. Marth, S. T. Sherry, G. McVean, and R. Durbin. The variant call format and VCFtools. *Bioinformatics*, 27(15):2156–2158, Aug. 2011. ISSN 1367-4803. doi: 10.1093/bioinformatics/btr330. URL https://academic.oup.com/bioinformatics/article/27/15/2156/402296. Publisher: Oxford Academic.

[10] X. Dong. SCcaller README (https://github.com/biosinodx/SCcaller/tree/61629e8c39a5b9c0e0891bd Aug. 2018. URL https://github.com/biosinodx/SCcaller/tree/61629e8c39a5b9c0e0891bdff7a9785f5028beec. original-date: 2016-06-09T15:43:47Z.

[11] X. Dong, L. Zhang, B. Milholland, M. Lee, A. Y. Maslov, T. Wang, and J. Vijg. Accurate identification of single-nucleotide variants in whole-genome-amplified single cells. *Nature Methods*, 14(5):491–493, Mar. 2017. ISSN 1548-7091, 1548-7105. doi: 10.1038/nmeth.4227. URL http://www.nature.com/doifinder/10.1038/nmeth.4227.

[12] F. Eggenberger and G. Pólya. Über die Statistik verketteter Vorgänge. *ZAMM*

- *Journal of Applied Mathematics and Mechanics / Zeitschrift für Angewandte Mathematik und Mechanik*, 3(4):279–289, 1923. ISSN 1521-4001. doi: 10.1002/zamm.19230030407. URL https://onlinelibrary.wiley.com/doi/abs/10.1002/zamm.19230030407.

[13] B. Grüning, R. Dale, A. Sjödin, B. A. Chapman, J. Rowe, C. H. Tomkins-Tinch, R. Valieris, and J. Köster. Bioconda: sustainable and comprehensive software distribution for the life sciences. *Nature Methods*, 15(7):475–476, July 2018. ISSN 1548-7105. doi: 10.1038/s41592-018-0046-7. URL https://www.nature.com/articles/s41592-018-0046-7.

[14] J. Hoell, M. Gombert, S. Ginzel, S. Loth, P. Landgraf, V. Käfer, M. Streiter, A. Prokop, M. Weiss, R. Thiele, and A. Borkhardt. Constitutional Mismatch Repair-deficiency and Whole-exome Sequencing as the Means of the Rapid Detection of the Causative MSH6 Defect. *Klinische Pädiatrie*, 226(06/07):357–361, Nov. 2014. ISSN 0300-8630, 1439-3824. doi: 10.1055/s-0034-1389905. URL http://www.thieme-connect.de/DOI/DOI?10.1055/s-0034-1389905.

[15] Y. Hou, L. Song, P. Zhu, B. Zhang, Y. Tao, X. Xu, F. Li, K. Wu, J. Liang, D. Shao, and e. al. Single-Cell Exome Sequencing and Monoclonal Evolution of a JAK2\mbox-Negative Myeloproliferative Neoplasm. *Cell*, 148(5):873–885, Mar. 2012. doi: 10.1016/j.cell.2012.02.028. URL http://dx.doi.org/10.1016/j.cell.2012.02.028. bibtex: Hou2012.

[16] htslib. C library for high-throughput sequencing data formats. URL https://github.com/samtools/htslib.

[17] J. O. Irwin. A Distribution Arising in the Study of Infectious Diseases. *Biometrika*, 41(1/2):266–268, 1954. ISSN 0006-3444. doi: 10.2307/2333023. URL https://www.jstor.org/stable/2333023.

[18] N. L. Johnson, A. W. Kemp, and S. Kotz. *Univariate discrete distributions*. Wiley, Hoboken, N.J, 3rd ed edition, 2005. ISBN 978-0-471-27246-5.

[19] J. Köster. Rust-Bio: a fast and safe bioinformatics library. *Bioinformatics*, 32 (3):444–446, Feb. 2016. ISSN 1367-4803, 1460-2059. doi: 10.1093/bioinformatics/btv573. URL `http://bioinformatics.oxfordjournals.org/content/32/3/444`.

[20] J. Köster and S. Rahmann. Snakemake—a scalable bioinformatics workflow engine. *Bioinformatics*, 28(19):2520–2522, Oct. 2012. ISSN 1367-4803, 1460-2059. doi: 10.1093/bioinformatics/bts480. URL `http://bioinformatics.oxfordjournals.org/content/28/19/2520`.

[21] J. Kuipers, K. Jahn, B. J. Raphael, and N. Beerenwinkel. Single-cell sequencing data reveal widespread recurrence and loss of mutational hits in the life histories of tumors. *Genome Research*, Oct. 2017. ISSN 1549-5469. doi: 10.1101/gr.220707.117.

[22] J. Kuipers, M. A. Tuncel, P. Ferreira, K. Jahn, and N. Beerenwinkel. Single-cell copy number calling and event history reconstruction. *bioRxiv*, page 2020.04.28.065755, Apr. 2020. doi: 10.1101/2020.04.28.065755. URL `https://www.biorxiv.org/content/10.1101/2020.04.28.065755v1`. Publisher: Cold Spring Harbor Laboratory Section: New Results.

[23] J. Köster, L. J. Dijkstra, T. Marschall, and A. Schönhuth. Varlociraptor: enhancing sensitivity and controlling false discovery rate in somatic indel discovery. *Genome Biology*, 21(1):98, Apr. 2020. ISSN 1474-760X. doi: 10.1186/s13059-020-01993-6. URL `https://doi.org/10.1186/s13059-020-01993-6`.

[24] B. Li, W. Chen, X. Zhan, F. Busonero, S. Sanna, C. Sidore, F. Cucca, H. M. Kang, and G. R. Abecasis. A Likelihood-Based Framework for Variant Calling and De Novo Mutation Detection in Families. *PLoS Genet*, 8(10):e1002944, Oct. 2012.

doi: 10.1371/journal.pgen.1002944. URL http://dx.doi.org/10.1371/journal.pgen.1002944.

[25] H. Li. Aligning sequence reads, clone sequences and assembly contigs with BWA-MEM. *arXiv:1303.3997 [q-bio]*, Mar. 2013. URL http://arxiv.org/abs/1303.3997. arXiv: 1303.3997.

[26] H. Li, B. Handsaker, A. Wysoker, T. Fennell, J. Ruan, N. Homer, G. Marth, G. Abecasis, and R. Durbin. The Sequence Alignment/Map format and SAMtools. *Bioinformatics*, 25(16):2078–2079, Aug. 2009. ISSN 1367-4803. doi: 10.1093/bioinformatics/btp352. URL https://academic.oup.com/bioinformatics/article/25/16/2078/204688. Publisher: Oxford Academic.

[27] J. Ling, G. Zhuang, B. Tazon-Vega, C. Zhang, B. Cao, Z. Rosenwaks, and K. Xu. Evaluation of genome coverage and fidelity of multiple displacement amplification from single cells by SNP array. *MHR: Basic science of reproductive medicine*, 15 (11):739–747, Nov. 2009. ISSN 1360-9947. doi: 10.1093/molehr/gap066. URL https://academic.oup.com/molehr/article/15/11/739/1000481.

[28] M. A. Lodato, M. B. Woodworth, S. Lee, G. D. Evrony, B. K. Mehta, A. Karger, S. Lee, T. W. Chittenden, A. M. D'Gama, X. Cai, L. J. Luquette, E. Lee, P. J. Park, and C. A. Walsh. Somatic mutation in single human neurons tracks developmental and transcriptional history. *Science*, 350(6256):94–98, Oct. 2015. ISSN 0036-8075, 1095-9203. doi: 10.1126/science.aab1785. URL http://science.sciencemag.org/content/350/6256/94.

[29] L. J. Luquette, C. L. Bohrson, M. A. Sherman, and P. J. Park. Identification of somatic mutations in single cell DNA-seq using a spatial model of allelic imbalance. *Nature Communications*, 10(1):1–14, Aug. 2019. ISSN 2041-1723.

doi: 10.1038/s41467-019-11857-8. URL https://www.nature.com/articles/s41467-019-11857-8.

[30] M. Martin. Cutadapt removes adapter sequences from high-throughput sequencing reads. *EMBnet.journal*, 17(1):10–12, May 2011. ISSN 2226-6089. doi: 10.14806/ej.17.1.200. URL https://journal.embnet.org/index.php/embnetjournal/article/view/200. Number: 1.

[31] P. Müller, G. Parmigiani, C. Robert, and J. Rousseau. Optimal Sample Size for Multiple Testing: The Case of Gene Expression Microarrays. *Journal of the American Statistical Association*, 99(468):990–1001, Dec. 2004. ISSN 0162-1459, 1537-274X. doi: 10.1198/016214504000001646. URL http://www.tandfonline.com/doi/abs/10.1198/016214504000001646.

[32] P. Müller, G. Parmigiani, and K. Rice. FDR and Bayesian multiple comparisons rules. *Proceedings of the ISBA 8th World Meeting on Bayesian Statistics (Valencia)*, June 2006. URL http://www.bepress.com/jhubiostat/paper115.

[33] R. C. NCBI. Database resources of the National Center for Biotechnology Information. *Nucleic Acids Research*, 44(D1):D7–D19, Jan. 2016. ISSN 0305-1048. doi: 10.1093/nar/gkv1290. URL https://academic.oup.com/nar/article/44/D1/D7/2503096.

[34] E. Neuwirth. RColorBrewer: ColorBrewer Palettes, Dec. 2014. URL https://cran.r-project.org/web/packages/RColorBrewer/index.html.

[35] M. Okabe and K. Ito. Color Universal Design (CUD) / Colorblind Barrier Free, Sept. 2008. URL https://jfly.uni-koeln.de/color/.

[36] B. S. Pedersen and A. R. Quinlan. Mosdepth: quick coverage calculation for genomes and exomes. *Bioinformatics*, 34(5):867–868, Mar. 2018. ISSN 1367-

4803. doi: 10.1093/bioinformatics/btx699. URL https://academic.oup.com/bioinformatics/article/34/5/867/4583630. Publisher: Oxford Academic.

[37] G. Peng, Y. Fan, T. B. Palculict, P. Shen, E. C. Ruteshouser, A.-K. Chi, R. W. Davis, V. Huff, C. Scharfe, and W. Wang. Rare variant detection using family-based sequencing analysis. *Proceedings of the National Academy of Sciences*, 110(10): 3985–3990, Mar. 2013. ISSN 0027-8424, 1091-6490. doi: 10.1073/pnas.1222158110. URL http://www.pnas.org/content/110/10/3985.

[38] G. Peng, Y. Fan, and W. Wang. FamSeq: A Variant Calling Program for Family-Based Sequencing Data Using Graphics Processing Units. *PLoS Comput Biol*, 10 (10):e1003880, Oct. 2014. doi: 10.1371/journal.pcbi.1003880. URL http://dx.doi.org/10.1371/journal.pcbi.1003880.

[39] Á. J. Picher, B. Budeus, O. Wafzig, C. Krüger, S. García-Gómez, M. I. Martínez-Jiménez, A. Díaz-Talavera, D. Weber, L. Blanco, and A. Schneider. TruePrime is a novel method for whole-genome amplification from single cells based on *Tth*PrimPol. *Nature Communications*, 7:13296, Nov. 2016. ISSN 2041-1723. doi: 10.1038/ncomms13296. URL https://www.nature.com/articles/ncomms13296.

[40] P. J. Renwick, J. Trussler, E. Ostad-Saffari, H. Fassihi, C. Black, P. Braude, C. M. Ogilvie, and S. Abbs. Proof of principle and first cases using preimplantation genetic haplotyping – a paradigm shift for embryo diagnosis. *Reproductive BioMedicine Online*, 13(1):110–119, Jan. 2006. ISSN 1472-6483. doi: 10.1016/S1472-6483(10)62024-X. URL http://www.sciencedirect.com/science/article/pii/S147264831062024X.

[41] rust htslib. HTSlib bindings and a high level Rust API for reading and writing BAM files. URL https://github.com/rust-bio/rust-htslib.

[42] G. Satas, S. Zaccaria, G. Mon, and B. J. Raphael. SCARLET: Single-cell tumor

phylogeny inference with copy-number constrained mutation losses. *Cell Systems*, 10(4):323–332.e8, Apr. 2020. ISSN 2405-4720. doi: 10.1016/j.cels.2020.04.001.

[43] S. T. Sherry, M.-H. Ward, M. Kholodov, J. Baker, L. Phan, E. M. Smigielski, and K. Sirotkin. dbSNP: the NCBI database of genetic variation. *Nucleic Acids Research*, 29(1):308–311, Jan. 2001. ISSN 0305-1048. doi: 10.1093/nar/29.1.308. URL `https://academic.oup.com/nar/article/29/1/308/1116004`.

[44] J. Singer, J. Kuipers, K. Jahn, and N. Beerenwinkel. Single-cell mutation identification via phylogenetic inference. *Nature communications*, 9(1):5144–5144, 2018. ISSN 2041-1723. doi: 10.1038/s41467-018-07627-7. URL `https://europepmc.org/articles/PMC6279798/`.

[45] C. Spits, C. Le Caignec, M. De Rycke, L. Van Haute, A. Van Steirteghem, I. Liebaers, and K. Sermon. Whole-genome multiple displacement amplification from single cells. *Nature Protocols*, 1(4):1965–1970, Nov. 2006. ISSN 1750-2799. doi: 10.1038/nprot.2006.326. URL `https://www.nature.com/articles/nprot.2007.326`.

[46] M. Taschuk and G. Wilson. Ten simple rules for making research software more robust. *PLOS Computational Biology*, 13(4):e1005412, Apr. 2017. ISSN 1553-7358. doi: 10.1371/journal.pcbi.1005412. URL `http://journals.plos.org/ploscompbiol/article?id=10.1371/journal.pcbi.1005412`.

[47] C. van Rijsbergen. Evaluation. In *Information Retrieval*, pages 112–140. Butterworths, London, 2 edition, 1979. URL `http://www.dcs.gla.ac.uk/Keith/Preface.html`.

[48] Y. Wang, J. Waters, M. L. Leung, A. Unruh, W. Roh, X. Shi, K. Chen, P. Scheet, S. Vattathil, H. Liang, A. Multani, H. Zhang, R. Zhao, F. Michor, F. Meric-Bernstam, and N. E. Navin. Clonal evolution in breast cancer revealed by single

nucleus genome sequencing. *Nature*, 512(7513):155–160, Aug. 2014. ISSN 0028-0836. doi: 10.1038/nature13600. URL `http://www.nature.com/nature/journal/v512/n7513/full/nature13600.html`.

[49] H. Wickham. *ggplot2: Elegant Graphics for Data Analysis*. Springer-Verlag New York, 2016. ISBN 978-3-319-24277-4. URL `https://ggplot2.tidyverse.org`.

[50] H. Wickham, M. Averick, J. Bryan, W. Chang, L. D. McGowan, R. François, G. Grolemund, A. Hayes, L. Henry, J. Hester, M. Kuhn, T. L. Pedersen, E. Miller, S. M. Bache, K. Müller, J. Ooms, D. Robinson, D. P. Seidel, V. Spinu, K. Takahashi, D. Vaughan, C. Wilke, K. Woo, and H. Yutani. Welcome to the tidyverse. *Journal of Open Source Software*, 4(43):1686, 2019. doi: 10.21105/joss.01686.

[51] X. Xu, Y. Hou, X. Yin, L. Bao, A. Tang, L. Song, F. Li, S. Tsang, K. Wu, H. Wu, and e. al. Single-Cell Exome Sequencing Reveals Single-Nucleotide Mutation Characteristics of a Kidney Tumor. *Cell*, 148(5):886–895, Mar. 2012. doi: 10.1016/j.cell.2012.02.025. URL `http://dx.doi.org/10.1016/j.cell.2012.02.025`. bibtex: Xu2012.

[52] T. W. Yee. The VGAM Package. *R News*, 8(2):28–39, Oct. 2008. URL `http://CRAN.R-project.org/doc/Rnews/`.

[53] T. W. Yee. *Vector Generalized Linear and Additive Models: With an Implementation in R*. Springer, New York, NY, USA, 2015.

[54] H. Zafar. MonoVar README (https://bitbucket.org/hamimzafar/monovar/src/7b47571d85a377c425f[illegible] URL `https://bitbucket.org/hamimzafar/monovar/src/7b47571d85a377c425feea86f6e591ee832fe342`.

[55] H. Zafar, Y. Wang, L. Nakhleh, N. Navin, and K. Chen. Monovar: single-nucleotide variant detection in single cells. *Nature Methods*, 13(6):505–507, June 2016. ISSN

1548-7105. doi: 10.1038/nmeth.3835. URL https://www.nature.com/articles/nmeth.3835.

[56] C. Zong, S. Lu, A. R. Chapman, and X. S. Xie. Genome-Wide Detection of Single-Nucleotide and Copy-Number Variations of a Single Human Cell. *Science*, 338 (6114):1622–1626, Dec. 2012. doi: 10.1126/science.1229164. URL http://dx.doi.org/10.1126/science.1229164.
