# Supplementary material for: Accurate and scalable variant calling from single cell DNA sequencing data with ProSolo
Source: Nat Commun. 2021 Nov 18;12:6744. doi: 10.1038/s41467-021-26938-w (PMC8602313; doi:10.1038/s41467-021-26938-w)
Supplement: Supplementary file 3 — Reporting Summary [file 41467_2021_26938_MOESM3_ESM.pdf]

nature research

Corresponding author(s): Alexander Schönhuth

Last updated by author(s): Sep 9, 2021

Reporting Summary

Nature Research wishes to improve the reproducibility of the work that we publish. This form provides structure for consistency and transparency in reporting. For further information on Nature Research policies, see our Editorial Policies and the Editorial Policy Checklist.

Statistics

For all statistical analyses, confirm that the following items are present in the figure legend, table legend, main text, or Methods section.

n/a	Confirmed	
☐	☒	The exact sample size (*n*) for each experimental group/condition, given as a discrete number and unit of measurement
☐	☒	A statement on whether measurements were taken from distinct samples or whether the same sample was measured repeatedly
☒	☐	The statistical test(s) used AND whether they are one- or two-sided<br>*Only common tests should be described solely by name; describe more complex techniques in the Methods section.*
☐	☒	A description of all covariates tested
☐	☒	A description of any assumptions or corrections, such as tests of normality and adjustment for multiple comparisons
☒	☐	A full description of the statistical parameters including central tendency (e.g. means) or other basic estimates (e.g. regression coefficient) AND variation (e.g. standard deviation) or associated estimates of uncertainty (e.g. confidence intervals)
☒	☐	For null hypothesis testing, the test statistic (e.g. *F*, *t*, *r*) with confidence intervals, effect sizes, degrees of freedom and *P* value noted<br>*Give P values as exact values whenever suitable.*
☐	☒	For Bayesian analysis, information on the choice of priors and Markov chain Monte Carlo settings
☒	☐	For hierarchical and complex designs, identification of the appropriate level for tests and full reporting of outcomes
☒	☐	Estimates of effect sizes (e.g. Cohen's *d*, Pearson's *r*), indicating how they were calculated

Our web collection on statistics for biologists contains articles on many of the points above.

Software and code

Policy information about availability of computer code

Data collection	For data collection, only software running on the respective machines (i.e. no custom code) were used.
Data analysis	All code used in data analysis and preparation of the manuscript, alongside a description of necessary steps for reproducing results, can be found in a GitHub repository accompanying this manuscript: https://github.com/prosolo/benchmarking_prosolo<br>To ensure its long-term availability and make it citable, we have created a release of this repository and archived it on Zenodo: https://doi.org/10.5281/zenodo.3769115<br>Exact versions of software used are given in the Methods and Supplementary Methods text, or are given in Supplementary Table S2.

For manuscripts utilizing custom algorithms or software that are central to the research but not yet described in published literature, software must be made available to editors and reviewers. We strongly encourage code deposition in a community repository (e.g. GitHub). See the Nature Research guidelines for submitting code & software for further information.

Data

Policy information about availability of data

All manuscripts must include a data availability statement. This statement should provide the following information, where applicable:

- Accession codes, unique identifiers, or web links for publicly available datasets
- A list of figures that have associated raw data
- A description of any restrictions on data availability

Accessions for all sequencing data used are given in the accompanying GitHub repository, namely at: https://github.com/ProSolo/benchmarking_prosolo/tree/v3.0/analysis_pipelines#dataset
The only restriction to data availability is that the newly generated data requires application for controlled access at: https://www.ebi.ac.uk/ega/datasets/EGAD00001005929

Wherever code and results data were used for generating figures, both is available in the accompanying GitHub repository. For all overview graphics that were created manually, SVG vector graphic versions are available in that same repository. All of this can be found at: https://github.com/ProSolo/benchmarking_prosolo/tree/v3.0/manuscript

Field-specific reporting

Please select the one below that is the best fit for your research. If you are not sure, read the appropriate sections before making your selection.

☒ Life sciences ☐ Behavioural & social sciences ☐ Ecological, evolutionary & environmental sciences

For a reference copy of the document with all sections, see nature.com/documents/nr-reporting-summary-flat.pdf

Life sciences study design

All studies must disclose on these points even when the disclosure is negative.

Sample size	The only sample size used in this study was the number of single cells analysed per dataset. For the previously published data by Dong et al. (2017), this was limited to two single cells that were available alongside the very close ground truth bulk sample. However, these cells have a very close bulk sample that can serve as a ground truth and thus serve as a good evaluation of the calling quality. To further investigate variability of results, we extended this to a greater number of single cells, namely five, for the newly generated data. This demonstrated, that the calling accuracy does not vary much among cells, so that even this number of cells is sufficient to generalize results. For the dataset by Wang et al. (2014) that was added during peer review, a total of 32 cells were available, with 16 each from a normal and a tumor cell population. Within these datasets, precision and recall still varied very little across single cells (see Supplementary Figures S 10 and S 11). Thus, the results presented here are robust across datasets and across cells within a dataset, so a greater number of cells would not have added insight to the analyses performed here.
Data exclusions	For the selection of the five newly sequenced cells, we focused on cells where whole genome amplification resulted in a reasonable breadth of coverage (at least 15 out of 16 loci were shown to have been amplified via quantitative real-time PCR). While this implicitly excludes cells where amplification was less uniform, this is common practice in single cell DNA sequencing.
Replication	We tried to ensure replicability of results by including both existing datasets, one generated for similar validation analyses and one for a different analysis, and generated a further validation dataset ourselves. In our own dataset, we used a different cell type and a different type of ground truth. We would thus assume, that our results are not dependent upon cell type, type of ground truth or the laboratory that generates the data.
Randomization	We did not perform any statistical testing between two or more groups of single cells that would require a randomization of samples.
Blinding	We did not perform any statistical testing between two or more groups of single cells that would require a blinding of researchers to the type of single cells sampled.

Reporting for specific materials, systems and methods

We require information from authors about some types of materials, experimental systems and methods used in many studies. Here, indicate whether each material, system or method listed is relevant to your study. If you are not sure if a list item applies to your research, read the appropriate section before selecting a response.

Materials & experimental systems

n/a	Involved in the study	
☒	☐	Antibodies
☒	☐	Eukaryotic cell lines
☒	☐	Palaeontology and archaeology
☒	☐	Animals and other organisms
☐	☒	Human research participants
☒	☐	Clinical data
☒	☐	Dual use research of concern

Methods

n/a	Involved in the study	
☒	☐	ChIP-seq
☐	☒	Flow cytometry
☒	☐	MRI-based neuroimaging

Human research participants

Policy information about studies involving human research participants

Population characteristics	All details for the patient from whom the granulocytes were extracted are given in a previous publication (https://doi.org/10.1055/s-0034-1389905):<br>Hoell JI, Gombert M, Ginzel S, et al. Constitutional mismatch repair-deficiency and whole-exome sequencing as the means of the rapid detection of the causative MSH6 defect. Klin Padiatr. 2014;226(6-7):357-361. doi:10.1055/s-0034-1389905<br>To reiterate here: The patient was female, 15 years old with co-sanguine parents. An initial T-cell non-Hodgkin lymphoma at age 20 months was treated according to NHL-BFM 95. 12 years later, an invasive adenocarcinoma was diagnosed and

	removed surgically, with a constitutional mismatch repair-deficiency (deletion in MSH6 gene, exon 6) diagnosed as a probable mutational mechanism behind the repeated carcinogenesis. A relapse occurred shortly after.
Recruitment	Data for this patient was generated, as previously published data (see Population characteristics above) provided an independent dataset to generate germline ground truth genotype calls.
Ethics oversight	Ethics Committee of the Medical Faculty of the Heinrich Heine University Düsseldorf.

Note that full information on the approval of the study protocol must also be provided in the manuscript.

Flow Cytometry

Plots

Confirm that:

☒ The axis labels state the marker and fluorochrome used (e.g. CD4-FITC).

☒ The axis scales are clearly visible. Include numbers along axes only for bottom left plot of group (a 'group' is an analysis of identical markers).

☒ All plots are contour plots with outliers or pseudocolor plots.

☒ A numerical value for number of cells or percentage (with statistics) is provided.

Methodology

Sample preparation	Mononuclear cells were derived from peripheral blood by ficoll density centrifugation. CD3 cells were depleted by magnetic cell separation using CD3 MicroBeads (Miltenyi Biotec) and a MiniMACS™ Separator. Cells were stained with FACS antibodies (as recommended by the manusfacturer Beckman Coulter) for 1 hour on ice in the dark. Cells were washed twice with PBS/1%BSA/0,05% Sodium Azide. Stained with Dapi (1 µg/ml) and measured on a MoFloXDP cell sorter (Beckman Coulter).
Instrument	MoFlo XDP (Beckman-Coulter)
Software	Summit Software (5.1.0) for data acquisition<br>Kaluza 2.1 for data analysis
Cell population abundance	Sorting was performed on several million blood cells. 59 CD66b+ single granulocytes were isolated into separate wells for further single cell processing. Several thousand further granulocytes were retained for separate processing as a granulocyte bulk sample.
Gating strategy	Input cells are CD3-T cell depleted mononuclear cells from peripheral blood. FSC/SSC gating on granulocyte population, positive for marker CD66b-FITC, Dapi gating on live population, negative for B cell markers (CD20 APC A700, CD19 APC, IgD FITC).

☒ Tick this box to confirm that a figure exemplifying the gating strategy is provided in the Supplementary Information.
